# Diaporphasines E and F: New Polyketides from the Saprotrophic Fungus *Lachnum* sp. IW157 Growing on the Reed Grass *Phragmites communis*

**DOI:** 10.1021/acsomega.3c05984

**Published:** 2023-10-30

**Authors:** Kunthida Phutthacharoen, Syeda J. Khalid, Hedda Schrey, Kevin D. Hyde, Marc Stadler, Sherif S. Ebada

**Affiliations:** † Department of Microbial Drugs, Helmholtz Centre for Infection Research GmbH (HZI), Inhoffenstraße 7, Braunschweig 38124, Germany; ‡ Center of Excellence in Fungal Research, Mae Fah Luang University, Chiang Rai 57100, Thailand; § School of Science, Mae Fah Luang University, Chiang Rai 57100, Thailand; ⊥ Institute of Microbiology, Technische Universität Braunschweig, Spielmannstraße 7, Braunschweig 38106, Germany; ∥ Department of Pharmacognosy, Faculty of Pharmacy, Ain Shams University, Cairo 11566, Egypt

## Abstract

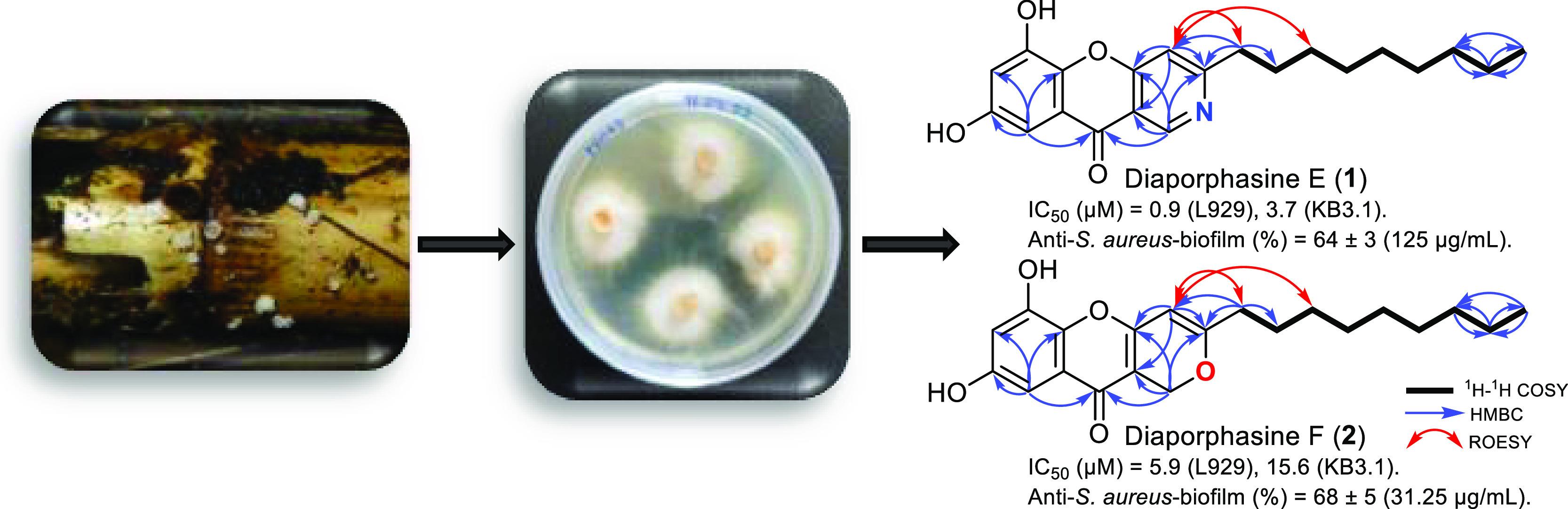

Chemical investigation for the mycelial extract of a saprotrophic fungus *Lachnum* sp. IW157 growing on the common reed grass *Phragmites communis* afforded the identification of two polyketide metabolites diaporphasines E (**1**) and F (**2**). Chemical structures of isolated compounds were unambiguously elucidated based on extensive 1D and 2D NMR spectral analyses in addition to their high-resolution mass spectrometry. The isolated compounds were assessed for their cytotoxicity and antimicrobial and biofilm inhibitory activities. While compound **1** revealed potent cytotoxicity against the tested cell lines L929 and KB3.1 with IC_50_ values of 0.9 and 3.7 μM, respectively, compound **2** exhibited moderate effects on the formation of *S. aureus* biofilms at 31.25 μg/mL.

## 1 Introduction

*Lachnum* Retz. is an ascomycete genus in Lachnaceae Raitv. featuring small apothecia that belongs to the order Helotiales and is typified by *Lachnum agaricinum* Retz. 1769. The apothecia of *Lachnum* species grow saprotrophically on various plant substrates such as ferns, grasses, rotting wood, or broadleaved trees. Till 2004, the genus was classified in the family Hyaloscyphaceae, but then Raitviir established the family Lachnaceae for *Lachnum* and other genera.^1^ This was verified by Hosoya et al. using a multigene phylogeny.^2^ Taxonomic position of the genus and family remains unchanged till present, with the Lachnaceae presently comprising 17 genera.^3^

The genus *Lachnum* has been identified in several reports to be a versatile source of fungal metabolites. Since the discovery of the chlorinated nematicidal metabolites, lachnumon and lachnumol A, from cultures of *Lachnum papyraceum*,^4^ a plethora of over 60 compounds were obtained from different species of the genus *Lachnum* that were previously undescribed including dihydroisocoumarin,^5,6^ chromone,^7^ and naphthalenone and phthalide derivatives.^8^

In addition, palmaenones A and B were reported from *L. palmae* as antimicrobial metabolites.^9^ Other reports disclosed the genus *Lachnum* as a producer of tetramic acid derivatives.^10^ However, *Lachnum* still represents an understudied genus in both taxonomy and secondary metabolites when compared to others.

In this study and in the outline of our major research focus to search for new fungal metabolites with potential antimicrobial or cytotoxic activity, we have explored the mycelial extract of a submerged culture fermentation of *Lachnum* sp. IW157 that was growing saprotrophically on the common reed grass *Phragmites communis*. The current paper is dedicated to the description of the isolation and characterization of two previously undescribed polyketide derivatives that were obtained from this culture.

## 2 Results and Discussion

Compound **1** was isolated as a yellowish brown amorphous solid with a molecular formula established to be C_21_H_25_NO_4_ based on HR-ESI-MS that revealed a protonated molecule at *m*/*z* 356.1858 [M + H]^+^ (calculated 356.1856) indicating ten degrees of unsaturation. The ^13^C NMR spectral data of **1** (Table 1) revealed the presence of 21 different carbon resonances that were distinguished into eight quaternary carbon atoms including one carbonyl carbon atom (δ_C_ 173.7, 166.7, 160.7, 155.7, 151.1, 144.7, 115.0, 113.7), four tertiary carbon atoms including a highly deshielded one (δ_C_ 148.7, 110.3, 108.0, 102.7), eight secondary carbon atoms (δ_C_ 37.6, 31.3, 28.92, 28.85, 28.8, 28.7, 28.6, 22.1), and a primary carbon atom (δ_C_ 14.0).

**Table 1:** ^1^H and ^13^C NMR Data of **1** and **2**

	**1**		**2**	
**pos.**	**δ**_**C**_**,**^a^**^,^**^c^**type**	**δ**_**H**_^b^**(multi,***J***[Hz])**	**δ**_**C**_**,**^b^**^,^**^c^**type**	**δ**_**H**_^b^**(multi,***J***[Hz])**
1	148.7,CH	9.13(s,1H)	63.8,CH2	5.11(s,2H)
3	166.7,C		169.5,C	
4	110.3,CH	7.40(brs)	94.2,CH	5.63(s,1H)
4a	160.7,C		158.0,C	
5a	144.7,C		144.5,C	
6	155.7,C		152.2,C	
7	102.7,CH	6.84(brs,1H)	102.5,CH	6.78(brs,1H)
8	151.1,C		150.0,C	
9	108.0,CH	7.39(s,1H)	107.2,CH	7.22(s,1H)
9a	113.7,C		109.0,C	
10	173.7,CO		171.7,CO	
10a	115.0,C		101.1,C	
11	37.6,CH2	2.82(t,7.5,2H)	33.0,CH2	2.24(t,7.5,2H)
12	28.8,CH2	1.70(q,7.5,2H)	25.8,CH2	1.50(m,2H)
13	28.7,CH2	1.28(m,2H)	28.0–29.1,CH2	1.22–1.30(m,overlapped,6H)
14	28.85,CH2	1.20–1.25(m,overlapped,6H)	28.0–29.1,CH2	1.22–1.30(m,overlapped,6H)
15	28.6,CH2	1.20–1.25(m,overlapped,6H)	28.0–29.1,CH2	1.22–1.30(m,overlapped,6H)
16	28.92,CH2	1.20–1.25(m,overlapped,6H)	28.0–29.1,CH2	1.22–1.30(m,overlapped,6H)
17	31.3,CH2	1.22(m,overlapped,2H)	31.0,CH2	1.24(m,overlapped,2H)
18	22.1,CH2	1.26(m,overlapped,2H)	22.1,CH2	1.25(m,overlapped,2H)
19	14.0,CH3	0.83(t,6.8,3H)	13.7,CH3	0.84(t,6.8,3H)

a Measured in DMSO-*d*_*6*_ at 125 MHz.

b Measured in DMSO-*d*_*6*_ at 500 MHz.

c Assigned based on HMBC and HSQC spectra.

The ^1^H NMR spectral data of **1** (Table 1, Figure S3) disclosed the presence of four aromatic protons distinguished into two deshielded aromatic protons at δ_H_ 9.13 and δ_H_ 7.40 that were correlated by the HSQC spectrum to two olefinic carbons at δ_C_ 148.7 and δ_C_ 110.3, respectively, suggesting the presence of an *ortho*-*para-*trisubstitued pyridine moiety in its structure.^11−14^ In addition, the ^1^H NMR spectrum of **1** revealed two more aromatic protons at δ_H_ 7.39 and δ_H_ 6.84 that were directly correlated in the HSQC spectrum to two carbon atoms at δ_C_ 108.0 and δ_C_ 102.7, respectively. The ^1^H–^1^H COSY spectrum of **1** (Figure 2, Figure S5) unveiled the existence of a key correlation extending from a methylene group at δ_H_ 2.82 (t, *J* = 7.5 Hz, H_2_-11) over seven methylene groups at δ_H_ 1.70 (q, *J* = 7.5 Hz, H_2_-12) and δ_H_ 1.28 (m, H_2_-13), three methylene groups at δ_H_ 1.20–1.25 (m, H_2_-14 to H_2_-16), δ_H_ 1.22 (m, H_2_-17), and δ_H_ 1.26 (m, H_2_-18), and a methyl triplet at δ_H_ 0.83 (t, *J* = 6.8 Hz, H_3_-19), suggesting the presence of a *n*-nonyl aliphatic side chain.

**Figure 1:**
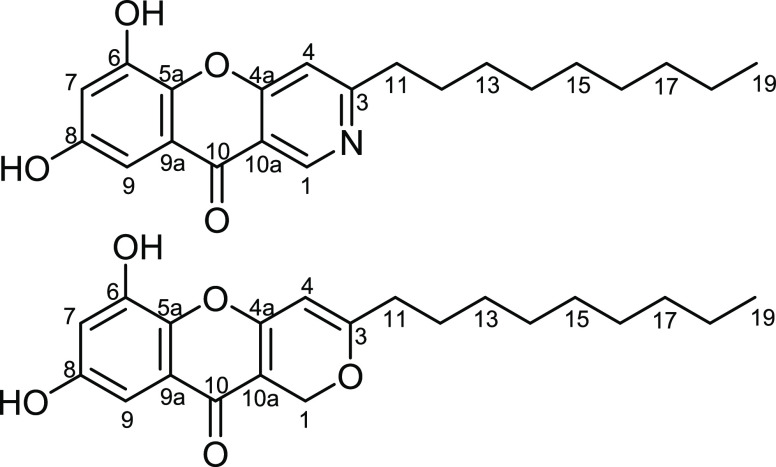
Structures of diaporphasines E (**1**) and F (**2**).

**Figure 2:**
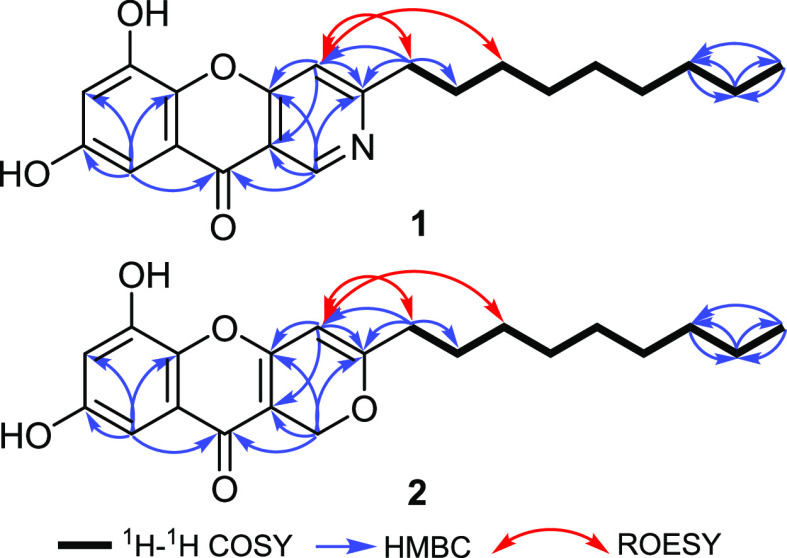
Key COSY, HMBC, and ROESY correlations of **1** and **2**.

Based on the obtained results and by searching the reported literature, compound **1** was suggested to be a polyketide derivative comprising a chromeno[3,2-*c*]pyridine skeleton in its structure, related to diaporphasines A–D and 7-hydroxy-8-methoxy-3-methyl-10-oxo-10*H*-chromeno[3,2-*c*]-pyridine-9-carboxylic acid that were reported as cytotoxic and antimicrobial metabolites from the mangrove-derived fungus *Diaporthe phaseolorum*(11) and the soil-derived fungus *Penicillium* sp.,^12^ respectively. To further confirm the depicted structure of **1** (Figure 1), the HMBC spectrum was recorded that revealed key correlations (Figure 2) from H-4 and H_2_-11 to a quaternary carbon at δ_C_ 166.7 (C-3), confirming the attachment of a *n*-nonyl side chain at C-3 in addition to the key HMBC correlations from two singlet aromatic protons H-1 at δ_H_ 9.13 and H-9 at δ_H_ 7.39 to a ketocarbonyl carbon atom at δ_C_ 173.7 (C-10). The latter revealed further HMBC correlations to three carbon resonances at δ_C_ 151.1 (C-8), δ_C_ 144.7 (C-5a), and δ_C_ 102.7 (C-7). Further confirmation to the position of the *n*-nonyl aliphatic side chain was obtained by the ROESY spectrum that displayed key ROE correlations from H-4 to H_2_-11 and H_2_-13. Based on the obtained results and by comparison with the reported literature, compound **1** was identified as a new chromeno[3,2-*c*]pyridine derivative that was given a trivial name diaporphasine E.

Compound **2** was obtained as a yellowish brown amorphous solid. Its molecular formula was determined to be C_21_H_26_O_5_ as revealed by HR-ESI-MS that disclosed a protonated molecule at *m*/*z* 359.1850 [M + H]^+^ (calculated 359.1853), indicating nine degrees of unsaturation. By comparing the two molecular formulas of **1** and **2**, it was obvious that the nitrogen atom in **1** was replaced by additional oxygen and hydrogen in **2**. This notion was undoubtedly interpreted by comparing the ^1^H NMR spectral data of **1** and **2** (Table 1, Figures S3 and S11) in which the latter revealed the presence of one oxygenated methylene group at δ_H_ 5.11 (s, H_2_-1) and three olefinic protons at δ_H_ 7.22 (s, H-9), δ_H_ 6.78 (br s, H-7), and δ_H_ 5.63 (s, H-4) that were directly correlated via the HSQC spectrum (Figure S14) to four carbon atoms at δ_C_ 63.8, δ_C_ 107.2, δ_C_ 102.5, and δ_C_ 94.2, respectively. The ^1^H–^1^H COSY spectra of **2** (Figures 2, Figure S12), as in **1**, exhibited the presence of a *n*-nonyl side chain by revealing a spin system from a methylene group at δ_H_ 2.24 (t, *J* = 7.5 Hz, H_2_-11) and extending over seven methylene groups at δ_H_ 1.50 (m, H_2_-12) and four methylene groups at δ_H_ 1.22–1.30 (m, H_2_-13 to H_2_-16), δ_H_ 1.24 (m, H_2_-17), and δ_H_ 1.25 (m, H_2_-18) ending by a methyl triplet at δ_H_ 0.84 (t, *J* = 6.8 Hz, H_3_-19). Based on the obtained results and by comparison with diaporphasine E (**1**), compound **2** was suggested to be a polyketide metabolite comprising a chromeno[3,2-*c*]pyran skeleton. Further confirmation to the depicted structure of **2** (Figure 1) was provided by HMBC spectra (Figure 2 and Figure S13) that revealed key correlations from H-4 and H_2_-11 to a quaternary carbon at δ_C_ 169.5 (C-3), confirming the location of the *n*-nonyl side chain at C-3. In addition, the HMBC spectra of **2** (Figure 2) revealed key correlations from H-9 and H_2_-1 to a ketocarbonyl carbon at δ_C_ 171.7 (C-10) and from H-9 to C-8 (δ_C_ 150.0), C-5a (δ_C_ 144.5), and C-7 (δ_C_ 102.5). Based on the aforementioned results and by comparison with reported literature, compound **2** was deduced to be a novel polyketide chromeno[3,2-*c*]pyran derivative that was given a trivial name, diaporphasine F.

**Figure 3:**
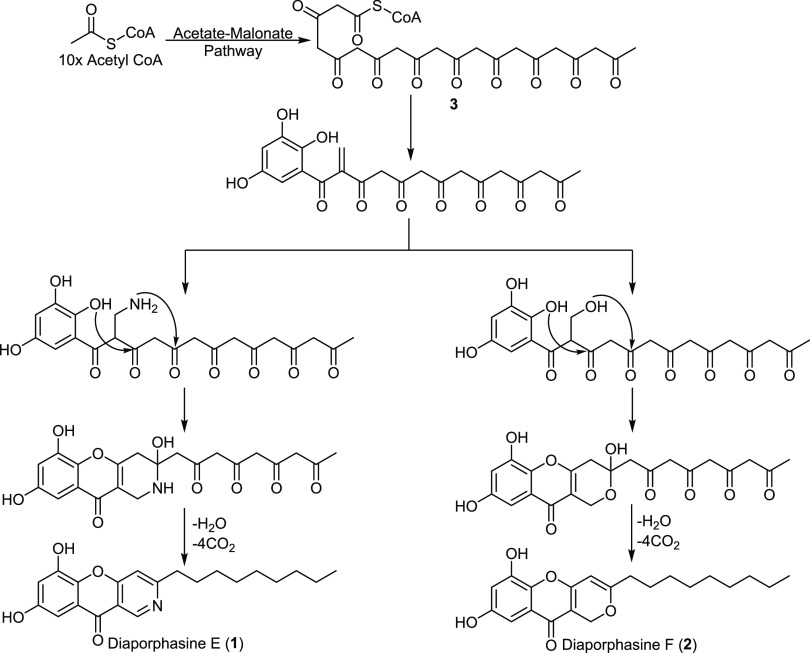
Plausible biosynthetic pathway for diaporphasines E (**1**) and F (**2**).

In terms of the core carbon skeleton and oxygenation pattern, diaporphasines E (**1**) and F (**2**) revealed structure similarity to other chromeno[3,2-*c*]pyridine derivatives such as diaporphasines A–D^11,12^ and fulvic acid,^12^ respectively. Therefore, they were suggested to be biosynthesized starting from a decaketide precursor (**3**, Figure 3), as previously suggested by Staunton and his fellows.^15,16^ At an intermediate phase of biosynthesis, transamination or hydroxylation will be accomplished followed by nucleophilic addition, condensation, and decarboxylation to afford either **1** or **2**.

All extracts derived from three small-scale cultures of *Lachnum* sp. IW157 were subjected to antimicrobial assay, and the obtained results (Table S1, Figures S19–S21) revealed that the mycelial extract of ZM culture had a moderate antimicrobial activity against *Bacillus subtilis* and *Escherichia coli* at a minimum inhibitory concentration of 75 μg/mL while a less potent activity was revealed against *Candida tenuis* at an MIC of 300 μg/mL. The isolated compounds, diaporphasines E (**1**) and F (**2**), were screened for their cytotoxicity (MTT) and antimicrobial assays. The obtained results (Table 2) revealed that diaporphasine E (**1**) possesses higher cytotoxic activities (IC_50_ values of 0.9 and 3.7 μM) compared to diaporphasine F (**2**) (IC_50_ values of 5.9 and 15.6 μM) against both tested cell lines, mouse fibroblasts (L929) and human endocervical adenocarcinoma (KB3.1), respectively.

**Table 2:** Cytotoxic Activity (IC_50_) of Compounds **1** and **2**

**IC**_**50**_**(μM)**			
**cell lines**	**1**	**2**	**epothilone B**
mouse fibroblast (L929)	0.9	5.9	6.5 × 10^–4^
uman endocervical adenocarcinoma (KB3.1)	3.7	15.6	1.73 × 10^–5^

In the antimicrobial activity assay against a panel of seven different microorganisms (Table S2), only diaporphasine F (**2**) exhibited weak activity against *B. subtilis* and *Staphylococcus aureus* with an MIC value of 33.3 μg/mL. Furthermore, diaporphasine E (**1**) showed weak inhibitory activity of 38% against the formation of *S. aureus* biofilms at a concentration 31.25 μg/mL whereas diaporphasine F (**2**) was slightly more active with inhibitory effects of 68% at the same concentration and inhibition of biofilms (27%) up to a concentration 7.8 μg/mL (Table 3). Interestingly, diaporphasine F (**2**), although less cytotoxic comparatively, showed potentially better antibiofilm activity.

**Table 3:** Inhibition of Biofilm Formation of *S. aureus* by Diaporphasines E (**1**) and F (**2**) at Different Concentrations^a^

**biofilm inhibition(% ± SD)**		
**tested train**	**1**	**2**
*Staphylococcus aureus* (DSM 1104)	64 ± 3 (125 μg/mL)	71 ± 11 (125 μg/mL)
	37 ± 11 (62.5 μg/mL)	68 ± 5 (31.25 μg/mL)
	38 ± 4 (31.25 μg/mL)	27 ± 9 (7.8 μg/mL)

a Reference: microporenic acid A (MAA); 80 ± 5 (125 μg/mL), 80 ± 5 (62.5 μg/mL), 78 ± 7 (31.25 μg/mL), 73 ± 9 (15.62 μg/mL), 44 ± 9 (7.8 μg/mL).

## 3 Conclusions

This study further supports the metabolic significance of the genus *Lachnum* as a prolific source of a vast array of metabolites where two uncommon polyketide metabolites (**1** and **2**) featuring a long C_9_-aliphatic side chain in their structures were reported in this study. Chemically, compounds **1** and **2** revealed structures related to the diaporphasines and proved to be unprecedentedly reported. In the conducted bioassays, diaporphasine E (**1**) exhibited more potent cytotoxic activity than diaporphasine F (**2**). However, only **2** revealed weak antimicrobial activity against *S. aureus* and *B. subtilis* as well as moderate inhibitory effects against formation of *S. aureus* biofilms. In conclusion, this study disclosed that the genus *Lachnum* is still a continuing source of new bioactive secondary metabolites that makes it worthy for further studies.

## 4 Materials and Methods

### 4.1 General Experimental Procedure

Optical rotations were determined using an MCP 150 polarimeter at 20 °C (Anton Paar OptoTec GmbH, Seelze, Germany). Ultraviolet–visible (UV/vis) spectra were obtained using a UV–vis spectrophotometer UV-2450 (Shimadzu, Kyoto, Japan). NMR spectra were recorded on Avance III 500 spectrometer (Bruker, Billerica, MA, USA, ^1^H NMR: 500 MHz, and ^13^C NMR: 125 MHz) dissolving compounds in deuterated DMSO-*d*_*6*_.

High-resolution electrospray ionization mass spectra (HR-ESI-MS) were acquired with an Agilent 1200 Infinity Series HPLC-UV system (Agilent Technologies, Santa Clara, California, USA) utilizing a C_18_ ACQUITY UPLC BEH column (2.1 × 50 mm, 1.7 μm: Waters, Milford, Massachusetts, USA), solvent A: deionized water + 0.1% formic acid; solvent B: acetonitrile (MeCN) + 0.1% formic acid, gradient: 5% B for 0.5 min increasing to 100% B in 19.5 min, maintaining 100% B for 5 min, flow rate 0.6 mL min^–1^, UV/vis detection 190–600 nm) connected to a time-of-flight mass spectrometer (ESI-TOF-MS, maXis, Bruker, Billerica, Massachusetts, USA) (scan range 100–2500 *m*/*z*, rate 2 Hz, capillary voltage 4500 V, dry temperature 200 °C). Molecular formulas of the detected compounds were calculated using the SmartFormula algorithm of the Compass Data Analysis software (Bruker, version 4.4).

### 4.2 Fermentation, Extraction, and Isolation

#### 4.2.1 Fungal Material and Characterization of the Culture of *Lachnum* sp. IW157

The culture we studied was derived by plating the spore print of an apothecium on YM (malt extract 10 g/L, yeast extract 4 g/L, d-glucose 4 g/L, agar 20 g/L, pH 6.3 before autoclaving) plates from the following specimen: Germany, Thuringia, Sonneberg, direction Meilschnitz, left side across “Froschteich”, alt. 359 m, location 50°21′39″N, 11°7′33″E, on previous year’s lying dead stalk of *Phragmites communis* (Poaceae), April 22, 2022. The specimen was collected and cultured by Ingo Wagner and registered under the herbarium code IW157. The culture is deposited at the DSMZ collection (Braunschweig Germany) under the designation no. DSM 116717.

For molecular characterization of the strain, fungal DNA was extracted from mycelium growing in YM agar using the fungal gDNA Miniprep Kit EZ-10 Spin Column protocol (NBS Biologicals, Cambridgeshire, UK). The amplification of the ITS and LSU regions was performed as described previously.^17,18^ PCR products were purified and sequenced using the Sanger Cycle sequencing method at Microsynth Seqlab GmbH (Göttingen, Germany). The sequences were deposited on GenBank with the accession numbers OR335757 (ITS) and OR335766 (LSU). As explained in the Supporting Information, the specimen has a similar morphology as *Lachnum carneolum*, but the sequence data deviated from those of the latter species deposited in GenBank. Therefore, it may just as well represent a new species. However, the clarification of the taxonomy of this fungus goes well beyond the scope of this study and will be reported separately. Thus, in this study the strain is referred to as *Lachnum* sp. until further work including the study of type material can be carried out.

#### 4.2.2 Small-Scale Fermentation and Extraction

The fungus was cultivated in three different liquid media (YM 6.3 medium: 10 g/mL malt extract, 4 g/mL, yeast extract, 4 g/mL, d-glucose and pH = 6.3, Q6 1/2 medium: 10 g/mL glycerol, 2.5 g/mL d-glucose, 5 g/mL cotton seed flour and pH = 7.2; ZM 1/2 medium: 5 g/mL molasses, 5 g/mL oatmeal, 1.5 g/mL d-glucose, 4 g/mL sucrose, 4 g/mL mannitol, 0.5 g/mL edamin, ammonium sulfate 0.5 g/mL, 1.5 g/mL calcium carbonate and pH = 7.2).^19^ The level of free glucose was tested daily by using Medi-Test glucose strips (Macherey-Nagel, Düren, Germany). The fermentation was terminated 3 days after glucose depletion, and the biomasses and supernatants were separated via vacuum filtration. The supernatants were extracted with equal amounts of ethyl acetate (200 mL) and filtered through anhydrous sodium sulfate. The resulting ethyl acetate extracts were evaporated to dryness in vacuum (rotary evaporator: Heidolph Instruments GmbH & Co. KG, Schwabach, Germany; pump: VacuuBrand GmbH & Co. KG, Wertheim am Main, Germany) at 40 °C. The mycelia were extracted with acetone (amounts equivalent to the original culture volume) in an ultrasonic bath (Sonorex Digital 10P, Bandelin Electronic GmbH & Co. KG, Berlin, Germany) at 40 °C for 30 min and filtered and the organic phase evaporated. The volume of the remaining aqueous phase was adjusted with an equal amount of distilled water and subjected to the same procedure as described for the supernatants.

#### 4.2.3 Scale-up Fermentation in Shake Flask Batches and Extraction

Preliminary results obtained from small-scale screening suggested that the fungus grew and produced best in ZM 1/2 medium. Moreover, the bioassay results on a small scale show that the extracts from the fungal culture in ZM 1/2 were active against *B. subtilis* and *E. coli*. Thus, a well-grown 14-day-old of *Lachnum* sp. IW157 in a YM agar plate was cut into small pieces using a 7 mm cork borer and five pieces inoculated in a 500 mL Erlenmeyer flask containing 200 mL of ZM 1/2 medium as a preculture. The preculture was preincubated at 23 °C on a shaker rotary at 140 rpm for 10 days and homogenized using Ultra-Turrax (T 25 easy clean digital, IKA), at 8000 rpm for 10 s. A 2 mL aliquot of homogenized substance was added in 200 mL of ZM 1/2 liquid medium in 500 mL for 25 flasks. Fermentation was aborted 3 days after the depletion of free glucose. The mycelia and supernatant from the batch fermentation were separated via vacuum filtration. The mycelia were extracted with acetone (amounts equivalent to the original culture volume) in an ultrasonic water bath at 40 °C for 30 min. The extracts were combined and the solvent evaporated *in vacuo* (40 °C). The remaining water phase was subjected to the same procedure as previously described for the mycelial fraction in small-scale extraction, repeating the extraction step three times and yielding 2.61 g. The supernatant (5 L) was extracted with an equal amount of ethyl acetate and filtered through anhydrous sodium sulfate. The resulting ethyl acetate extract was evaporated to dryness *in vacuo* to afford 1.86 g of extract.

#### 4.2.4 Isolation of Secondary Metabolites

The mycelium crude extract (2.61 g) was dispersed in 100 mL of MeCN/deionized water (3:7) and then subjected to liquid–liquid fractionation against 250 mL of *n*-heptane that results in a precipitate at the interface between two immiscible liquid phases. The precipitate was filtered and separately collected affording 170 mg of dry weight. The precipitate was then purified using preparative reversed-phase liquid chromatography (PLC 2020, Gilson, Middleton, Wisconsin, USA) implemented with a Gemini C_18_ column (250 × 21.2 mm, 10 μm, Phenomenex, Aschaffenburg, Germany) as a stationary phase. Deionized water (Milli-Q, Millipore, Schwalbach, Germany) supplemented with 0.1% formic acid (FA) (solvent A) and acetonitrile (MeCN) with 0.1% FA (solvent B) were used as the mobile phase. The elution gradient used for fractionation started using 20% of solvent B (MeCN + 0.1% FA) and 80% of solvent A (deionized water + 0.1% FA) for 5 min. Then, the gradient continued from 20 to 40% of solvent B over 10 min and from 40 to 100% of solvent B over 30 min ending by holding 100% of solvent B for 10 min. The flow rate was set to 15 mL/min, and UV detection was carried out at 210, 225, 275, and 330 nm. For this extract, 13 fractions (F1–F13) were selected according to the observed peaks and further analysis of the fractions using HPLC-MS revealed that the two fractions constituted pure compounds, fraction 5 (**1**, *t*_R_ = 30.0 min, 2.0 mg) and fraction 10 (**2**, *t*_R_ = 35.0 min, 1.5 mg).

##### 4.2.4.1 Diaporphasine E (**1**)

Yellowish brown amorphous solid; UV/vis (MeOH): λ_max_ (log ε) = 224 (1.1), 300 (0.2), 355 (0.1) nm; NMR data (^1^H NMR: 500 MHz, ^13^C NMR: 125 MHz in DMSO-*d*_*6*_), see Table 1; HR-(+)ESIMS: *m*/*z* 356.1858 [M + H]^+^ (calcd. 356.1856 for C_21_H_26_NO_4_^+^).

##### 4.2.4.2 Diaporphasine F (**2**)

Yellowish brown amorphous solid; UV/vis (MeOH): λ_max_ (log ε) = 224 (1.2), 348 (0.4) nm; NMR data (^1^H NMR: 500 MHz, ^13^C NMR: 125 MHz in DMSO-*d*_*6*_), see Table 1; HR-(+)ESIMS: *m*/*z* 359.1850 [M + H]^+^ (calcd. 359.1853 for C_21_H_27_O_5_^+^).

#### 4.2.5 Antimicrobial Assay

The antifungal and antibacterial activities (MIC in μg/mL) of all extracts and isolated compounds were determined in serial dilution assay as previously described.^19,20^

#### 4.2.6 Cytotoxicity Assay

*In vitro* cytotoxicity (IC_50_) assessments were carried out on the isolated compounds based on MTT (3-(4,5-dimethylthiayol-2-yl)-2,5-diphenyltetrazolium bromide) test in 96-well plates, using the cell lines L929 (mouse fibroblasts) and KB3.1 (human endocervical adenocarcinoma) in accordance with our previously established methods.^20,21^

#### 4.2.7 Biofilm Inhibitory Assay

Both diaporphasines E (**1**) and F (**2**) were evaluated for their inhibitory activity against *S. aureus* (DSM 1104) biofilms according to the protocol reported previously.^22^ In short, *S. aureus* was precultured from a −20 °C stock and incubated for 18 h. For the biofilm assay, OD_600_ of culture was adjusted in media suspension to 0.001 McFarland standard. The *S. aureus* biofilm was then allowed to grow along with serially diluted compounds (**1** and **2**: 125–1.9 μg/mL) for 24 h. Biofilm inhibition by **1** and **2** was assessed by subsequent steps of crystal violet staining and washing. Finally, the stained biomass was analyzed by a microtiter plate reader (Synergy 2, BioTek). Methanol was run as a solvent control, in which 100% was considered with no inhibition activity against *S. aureus* biofilms. Microporenic acid (MAA) was used as positive control.^23^ SD of two repeats with duplicates each was 15% or less.

## Supporting Information Available

1D (^1^H/^13^C) and 2D (^1^H–^1^H COSY, HMBC, HSQC and ROESY) spectra in addition to all HPLC-ESI-MS and HR-ESI-MS of diaporphasines E (**1**) and F (**2**) (PDF)
