# Supplementary material for: Diaporphasines E and F: New Polyketides from the Saprotrophic Fungus Lachnum sp. IW157 Growing on the Reed Grass Phragmites communis
Source: ACS Omega. 2023 Oct 30;8(44):41689–95. doi: 10.1021/acsomega.3c05984 (PMC10633876; doi:10.1021/acsomega.3c05984)
Supplement: Supplementary file 1 — ao3c05984_si_001.pdf [file ao3c05984_si_001.pdf]

Supporting Information for:

Diaporphasines E and F: New polyketides from the saprotrophic fungus *Lachnum* sp. IW157 growing on the reed grass *Phragmites communis*

Kunthida Phutthacharoen,[†,‡,§] Syeda J. Khalid,[†,⊥] Hedda Schrey,[†,⊥] Kevin D. Hyde,[‡,§] Marc Stadler,[†,⊥]* and Sherif S. Ebada,[†,∥]*

[†] Department of Microbial Drugs, Helmholtz Centre for Infection Research GmbH (HZI), Inhoffenstraße 7, 38124 Braunschweig, Germany

[‡] Center of Excellence in Fungal Research, Mae Fah Luang University, Chiang Rai 57100, Thailand

[§] School of Science, Mae Fah Luang University, Chiang Rai 57100, Thailand

[⊥] Institute of Microbiology, Technische Universität Braunschweig, Spielmannstraße 7, 38106 Braunschweig, Germany

[∥] Department of Pharmacognosy, Faculty of Pharmacy, Ain Shams University, 11566 Cairo, Egypt

* Corresponding authors: sherif.elsayed@helmholtz-hzi.de; sherif_elsayed@pharma.asu.edu.eg (S.S.E.); Marc.Stadler@helmholtz-hzi.de (M.S.); Tel.: +49-531-6181-4240; Fax +49-531-6181-9499

Abstract

Chemical investigation of the mycelial extract of a saprotrophic fungus *Lachnum* sp. IW157 growing on the common reed grass *Phragmites communis* afforded the identification of two polyketide metabolites diaporphasines E (**1**) and F (**2**). Chemical structures of isolated compounds were unambiguously elucidated based on extensive 1D and 2D NMR spectral analyses in addition to their high-resolution mass spectrometry. The isolated compounds were assessed for their cytotoxicity, antimicrobial and biofilm inhibitory activities. While compound **1** revealed potent cytotoxicity against the tested cell lines L929 and KB3.1 with IC_{50} values of 0.9 and 3.7 µM, respectively, compound **2** exhibited moderate effects on the formation of *S. aureus* biofilms at 31.25 μgmL^{-1}.

Contents of Supporting Information

#	Contents	Page
**1**	Figure S1. HPLC-LRESIMS of **1**.	**S3**
**2**	Figure S2. HPLC-HRESIMS of **1**.	**S4**
**3**	Figure S3. ^{1}H NMR spectrum of **1** in DMSO-d_6 at 500 MHz.	**S5**
**4**	Figure S4. ^{13}C NMR spectrum of **1** in DMSO-d_6 at 125 MHz.	**S6**
**5**	Figure S5. ^{1}H-^{1}H COSY spectrum of **1** in DMSO-d_6 at 500 MHz.	**S7**
**6**	Figure S6. HMBC spectrum of **1** in DMSO-d_6 at 500 MHz.	**S8**
**7**	Figure S7. HSQC spectrum of **1** in DMSO-d_6 at 500 MHz.	**S9**
**8**	Figure S8. ROESY spectrum of **1** in DMSO-d_6 at 500 MHz.	**S10**
**9**	Figure S9. HPLC-LRESIMS of **2**.	**S11**
**10**	Figure S10. HPLC-HRESIMS of **2**.	**S12**
**11**	Figure S11. ^{1}H NMR spectrum of **2** in DMSO-d_6 at 500 MHz.	**S13**
**12**	Figure S12. ^{1}H-^{1}H COSY spectrum of **2** in DMSO-d_6 at 500 MHz.	**S14**
**13**	Figure S13. HMBC spectrum of **2** in DMSO-d_6 at 500 MHz.	**S15**
**14**	Figure S14. HSQC spectrum of **2** in DMSO-d_6 at 500 MHz.	**S16**
**15**	Figure S15. ROESY spectrum of **2** in DMSO-d_6 at 500 MHz.	**S17**
**16**	Figure S16. Effects on the biofilm formation of *S. aureus* after 24 h treatment with diaporphasines E (**1**) and F (**2**). Microporenic acid A (MAA) was used as positive control. Methanol was used as a solvent control and taken as 100%. Error bars indicate standard deviation of duplicates in two biological repeats; p values: * $p < 0.05$, ** $p < 0.01$, *** $p < 0.001$.	**S18**
**17**	Figure S17. Maximum likelihood phylogenetic tree inferred from 177 taxa of Lachnaceae based on combined LSU and ITS sequence data. MLBP values ≥70% are given above the nodes. Strain/culture numbers are given after the taxon names. The tree is rooted with *Pezicula chiangraiensis* MFLUCC 15-0170 and *P. cinnamomea* CBS 100248. Newly generated sequence is in **purple**.	**S19**
**18**	Figure S18. a. *Lachnum* sp. IW157 growing on *Phragmites communis* (Poaceae) b. Culture of *Lachnum* sp. IW157 on YM media	**S20**
**19**	Table S1. Minimum inhibitory concentration (MIC) test for crude extract of *Lachnum* sp. IW157 (DSM 116717) against *Escherichia coli*, *Bacillus subtilis* and *Candida tenuis*.	**S21**
**20**	Figure S19. Shown MIC result of *Lachnum* sp. IW157 (DSM 116717) against *E. coli*.	**S21**
**21**	Figure S20. Shown MIC result of *Lachnum* sp. IW157 (DSM 116717) against *B. subtilis*.	**S22**
**22**	Figure S21. Shown MIC result of *Lachnum* sp. IW157 (DSM 116717) against *Candida tenuis*.	**S22**
**23**	Table S2. Minimum inhibitory concentration (MIC) of compounds **1** and **2**.	**S23**

Generic Display Report

Analysis Info

Analysis Name	S:\DATA\AmaZon\gph22_Kunthide-Gift Phutthacharoen\04-23\22045 ZM Mf2-F6_GB7_01_46507.d	Acquisition Date	28.04.2023 01:51:25
Method	46507.m	Operator	tti
Sample Name	22045 ZM Mf2-F6	Instrument	amaZon speed
Comment			

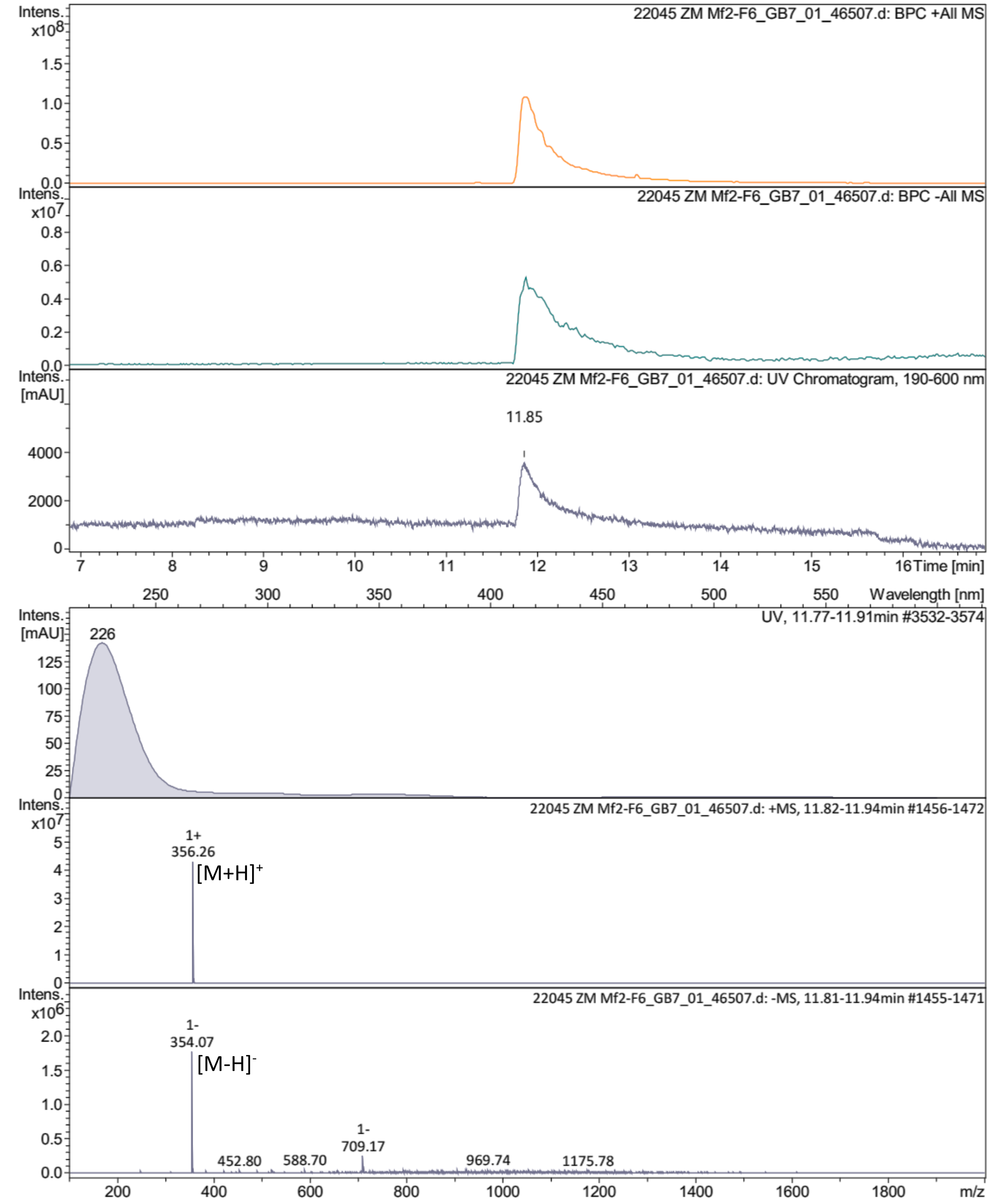

Bruker Compass DataAnalysis 4.4 printed: 14.07.2023 09:56:08 by: sel22 Page 1 of 1

Figure S1. HPLC-LRESIMS of **1**.

Generic Display Report

Analysis Info

Analysis Name	S:\DATA\MaXis\GPH22_Gift_Kunthide_Phutthacharoen\23_05\22045 ZM MF2-F5a_14_01_11430.d	Acquisition Date	03.05.2023 12:29:36
Method	pos_säure_10000_screening_ms_100_2500_line.m	Operator	ate06
Sample Name	22045 ZM MF2-F5a	Instrument	maXis
Comment	Screening01<br>Waters Acquity UPLC BEH C_18 1,7um 2.1x50mm		

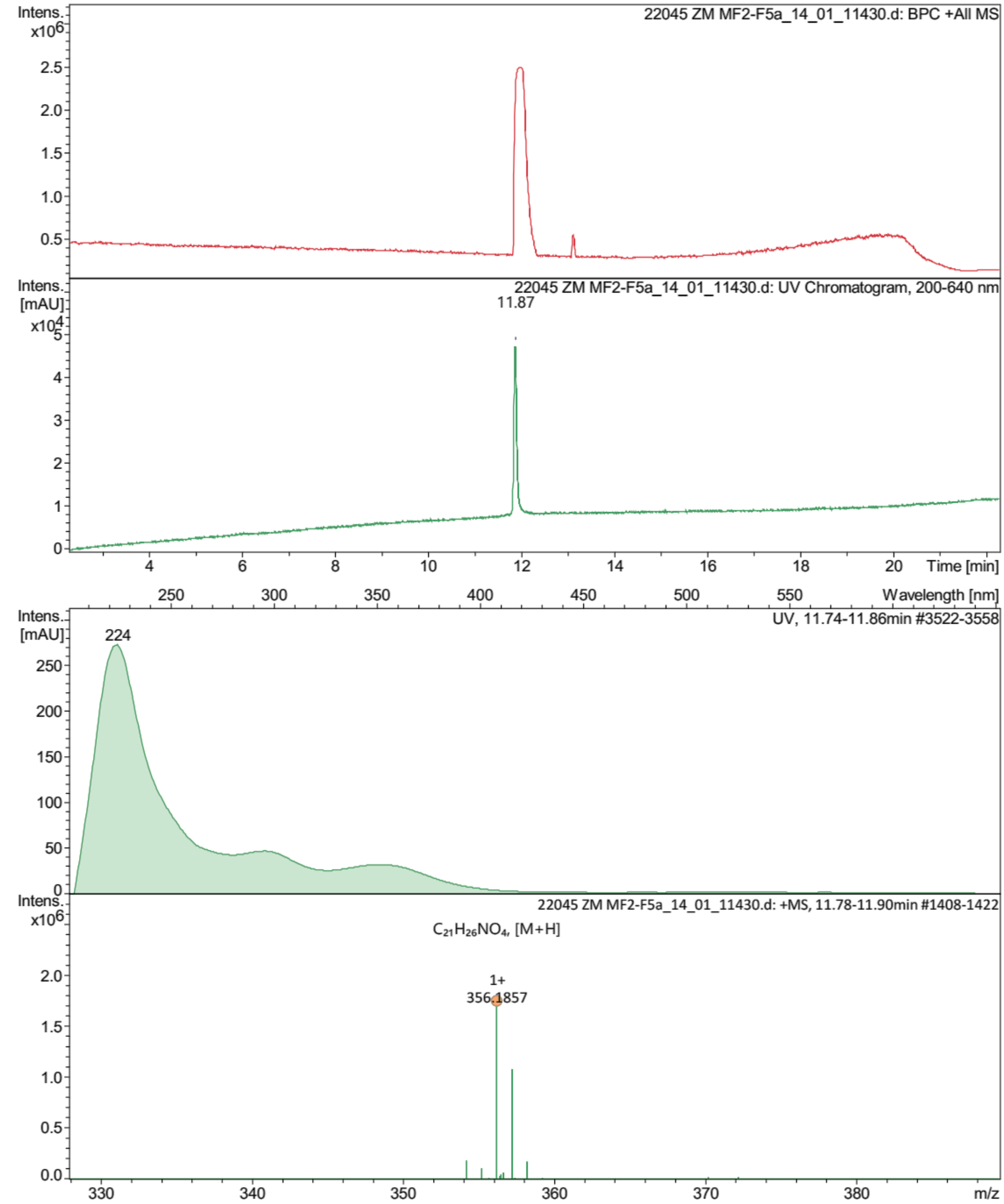

Bruker Compass DataAnalysis 4.4 printed: 14.07.2023 09:39:38 by: sel22 Page 1 of 1

Figure S2. HPLC-HRESIMS of **1**.

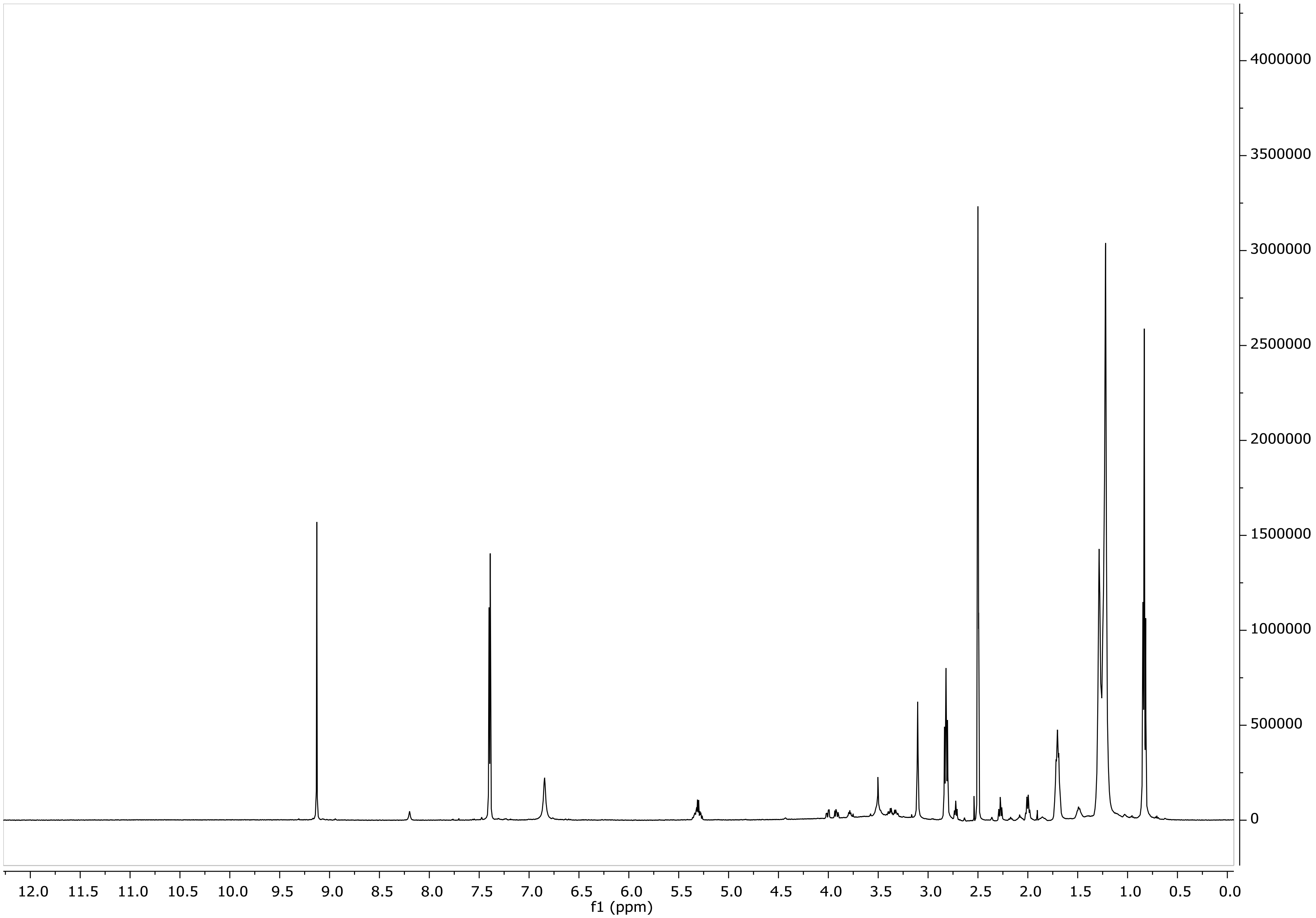

Figure S3. ^{1}H NMR spectrum of **1** in DMSO-d_6 at 500 MHz.

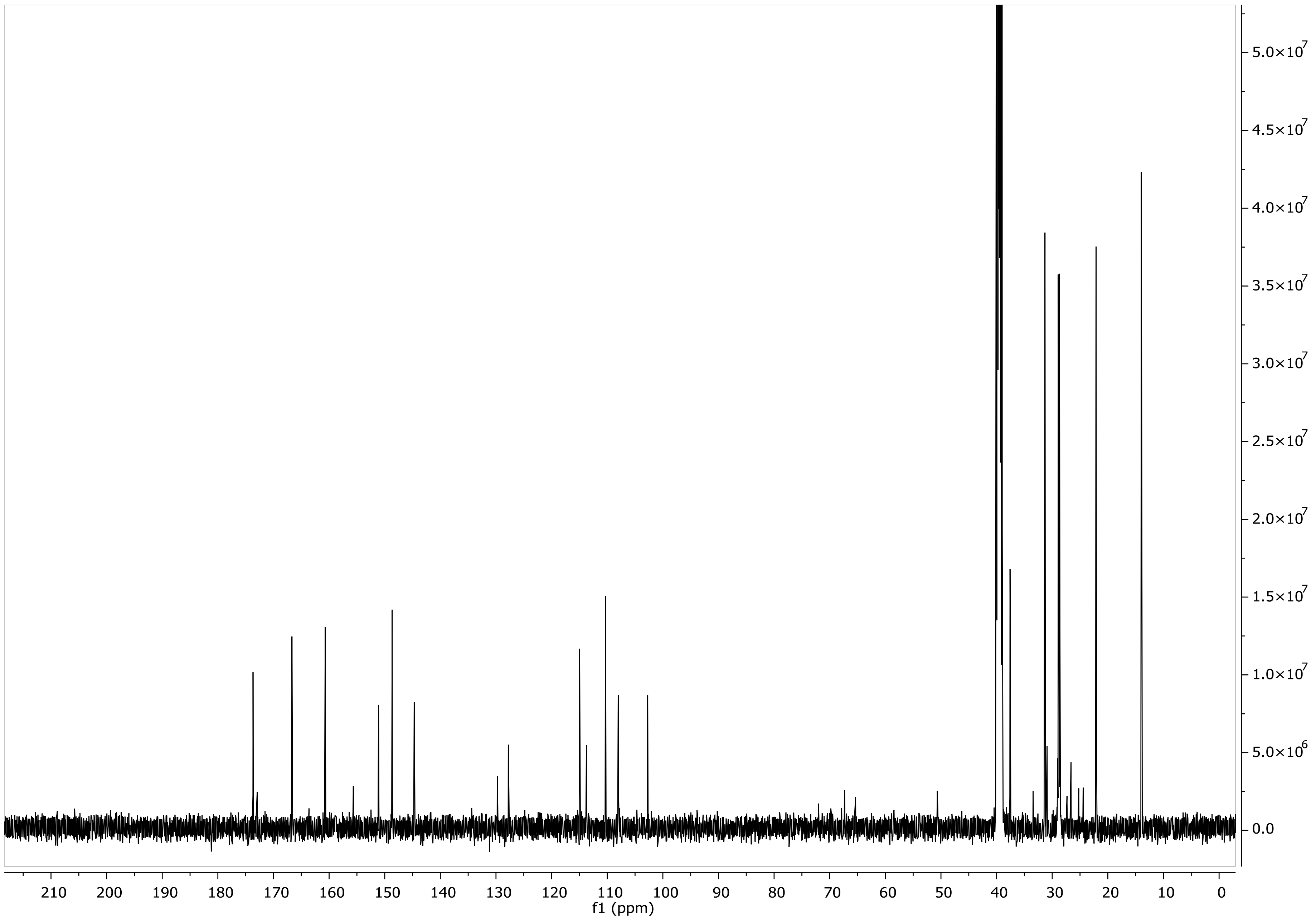

Figure S4. ^{13}C NMR spectrum of **1** in DMSO-d_6 at 125 MHz.

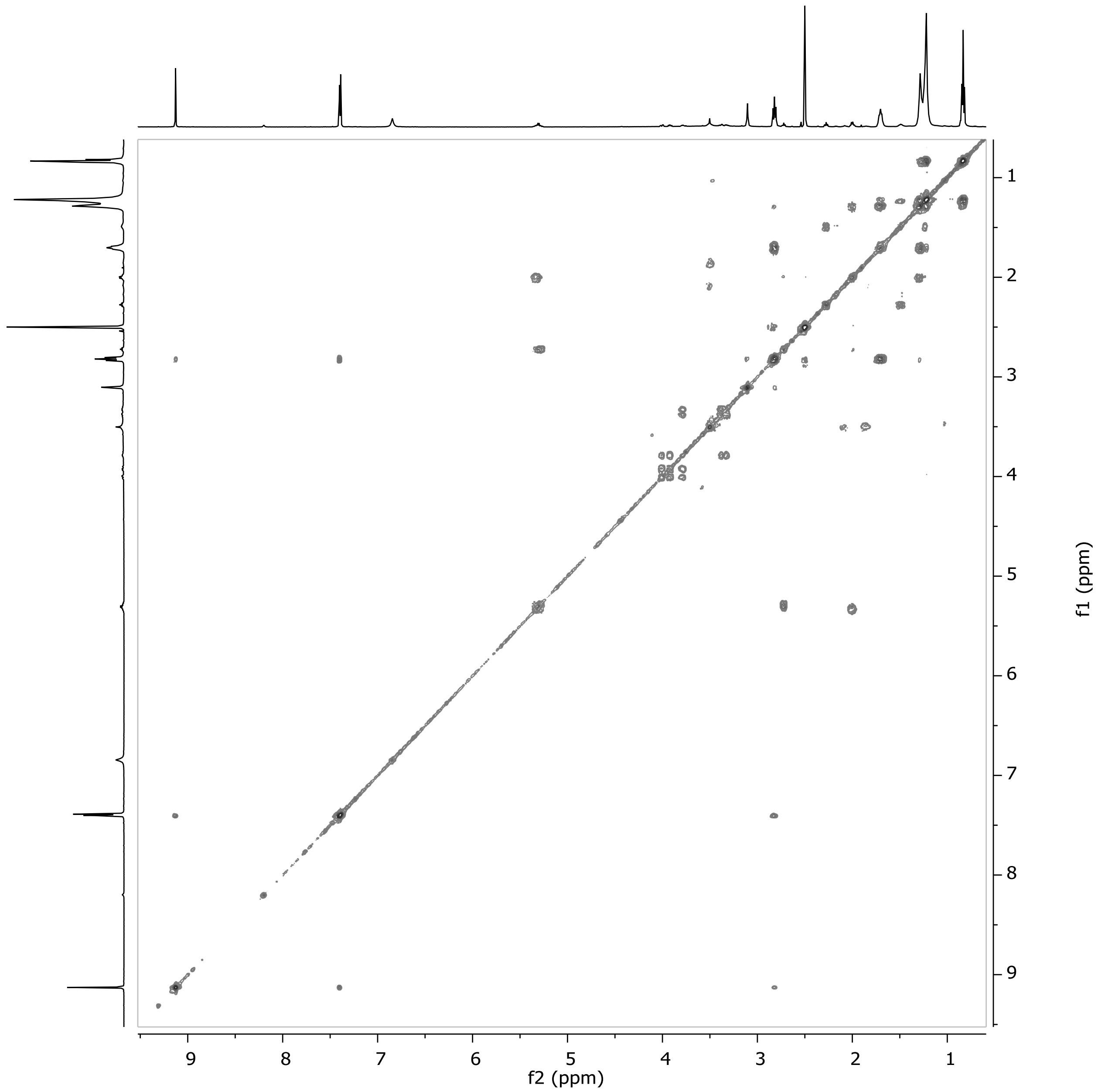

Figure S5. ^1H-^1H COSY spectrum of **1** in DMSO-d_6 at 500 MHz.

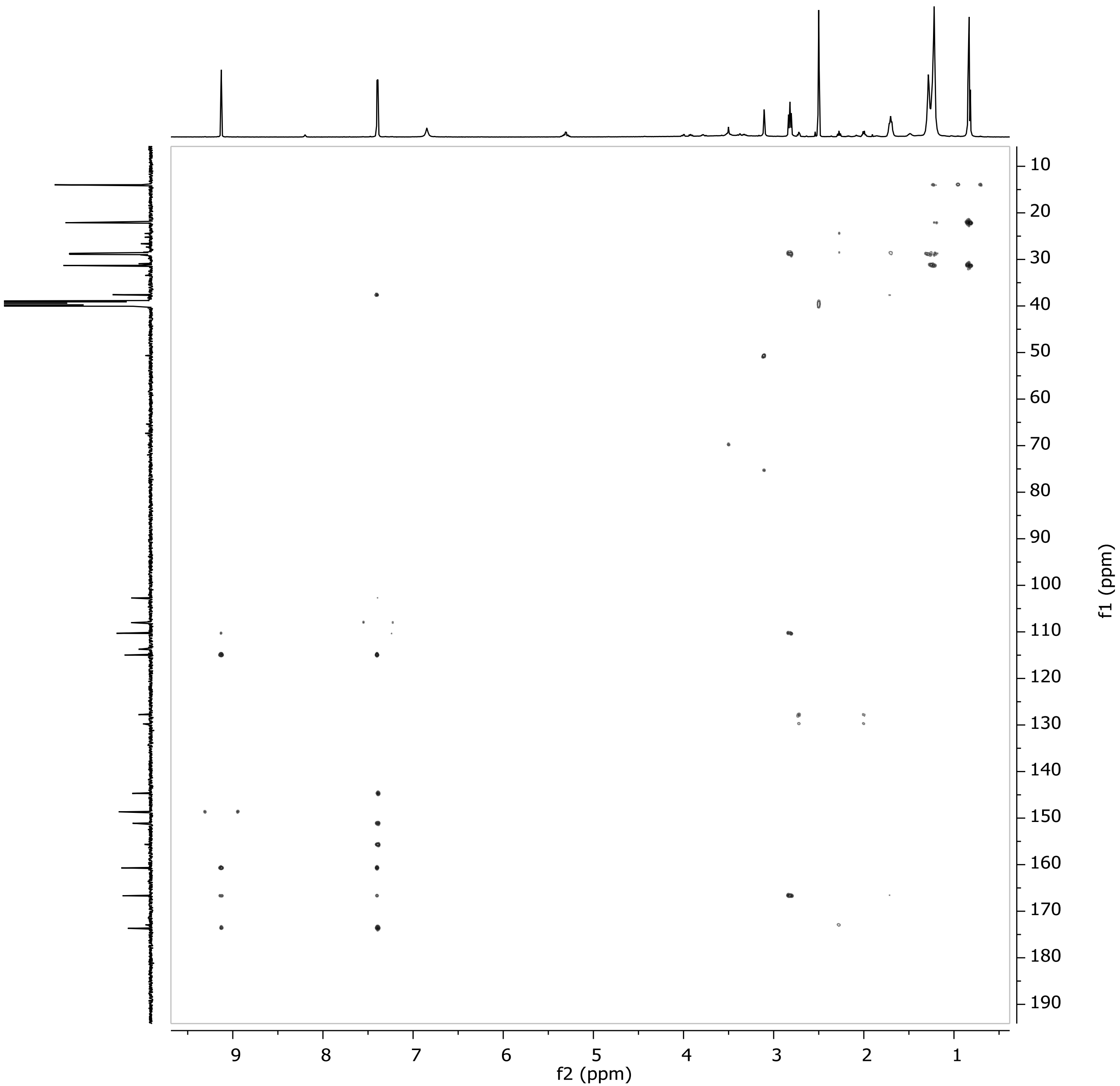

Figure S6. HMBC spectrum of **1** in DMSO-d_6 at 500 MHz.

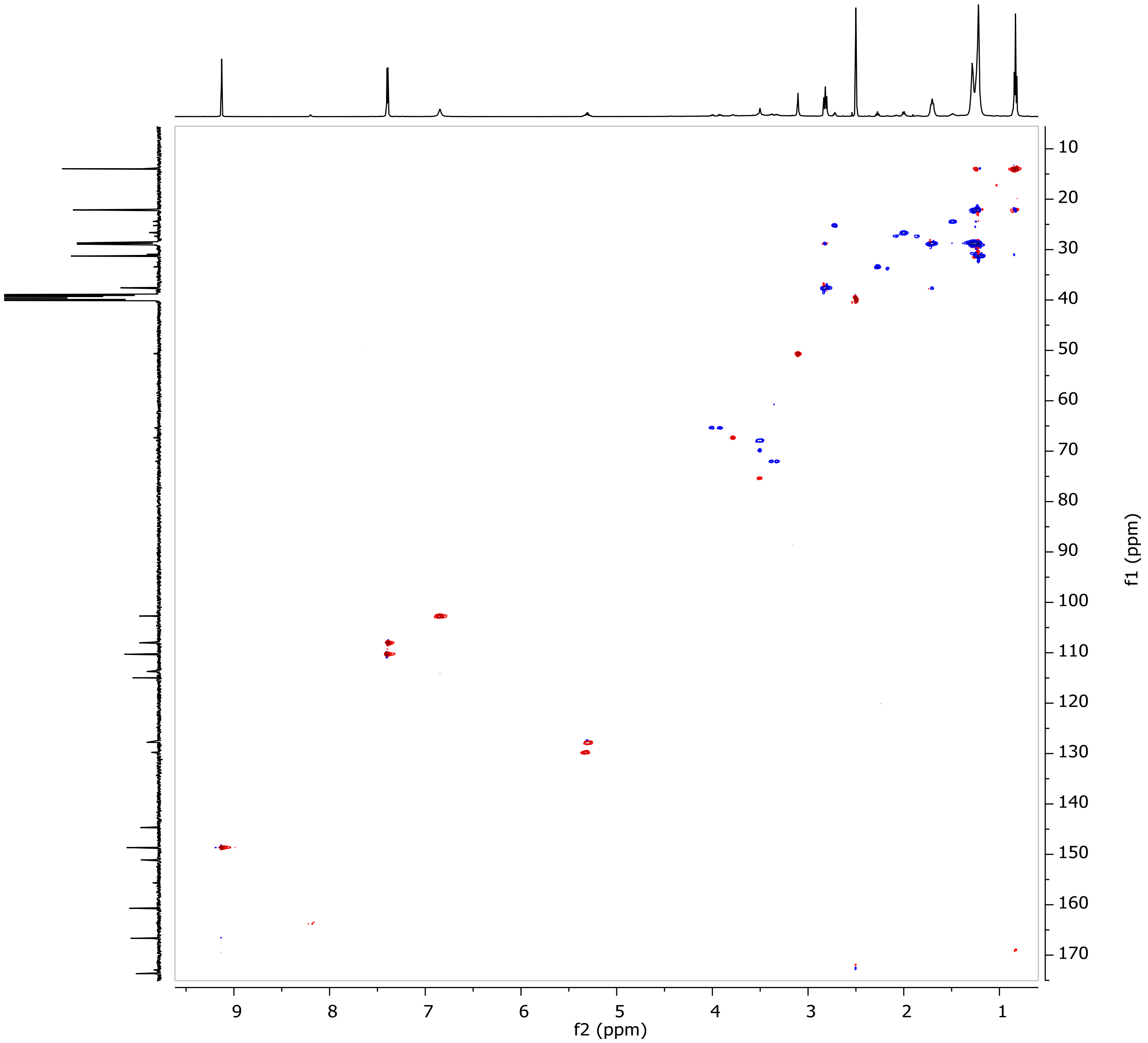

Figure S7. HSQC spectrum of **1** in DMSO-d_6 at 500 MHz.

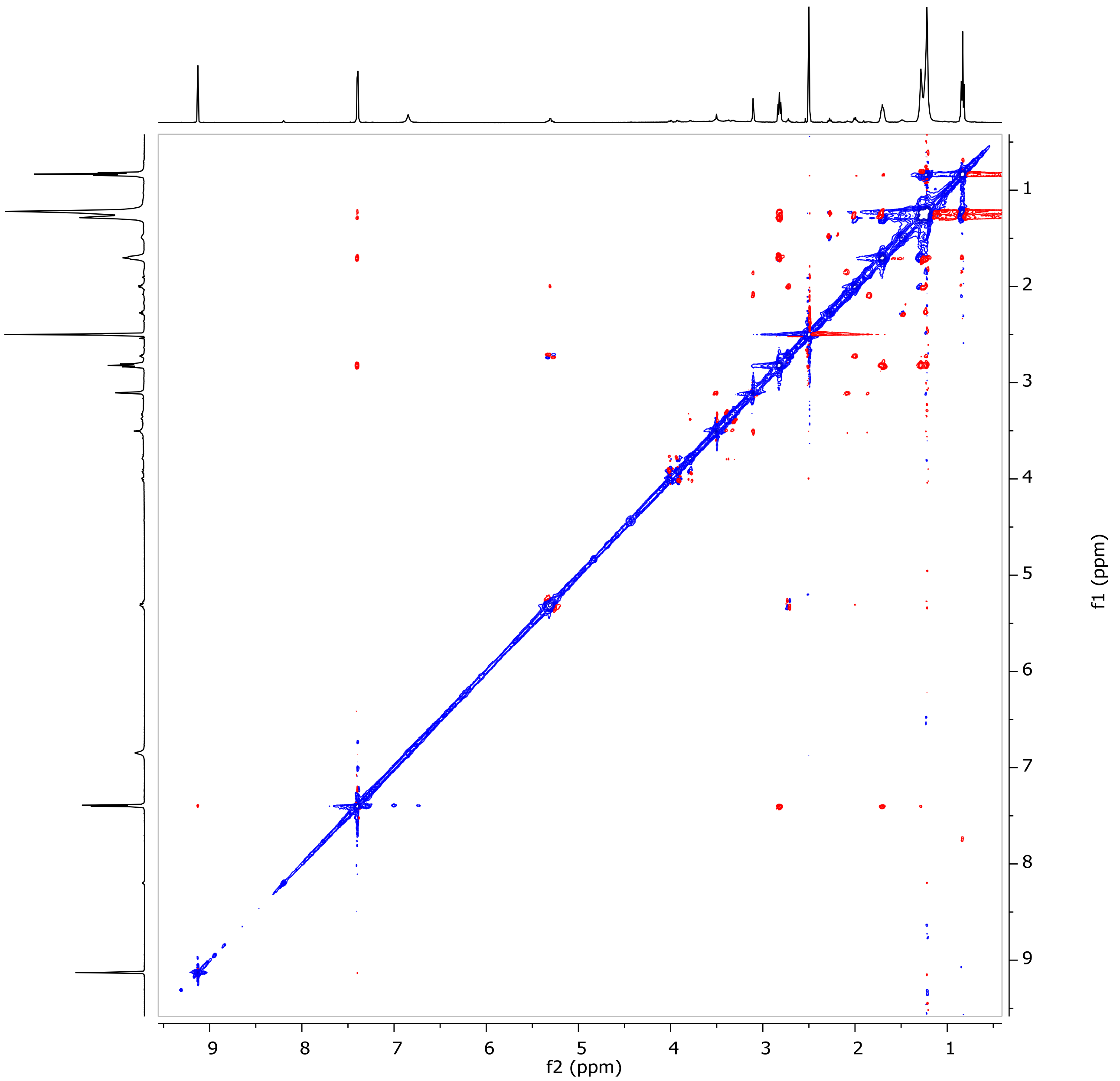

Figure S8. ROESY spectrum of **1** in DMSO-d_6 at 500 MHz.

Generic Display Report

Analysis Info

Analysis Name	S:\DATA\AmaZon\gph22_Kunthide-Gift Phutthacharoen\04-23\22045 ZM Mf2-F10_GC1_01_46509.d	Acquisition Date	28.04.2023 03:03:47
Method	46509.m	Operator	tti
Sample Name	22045 ZM Mf2-F10	Instrument	amaZon speed
Comment			

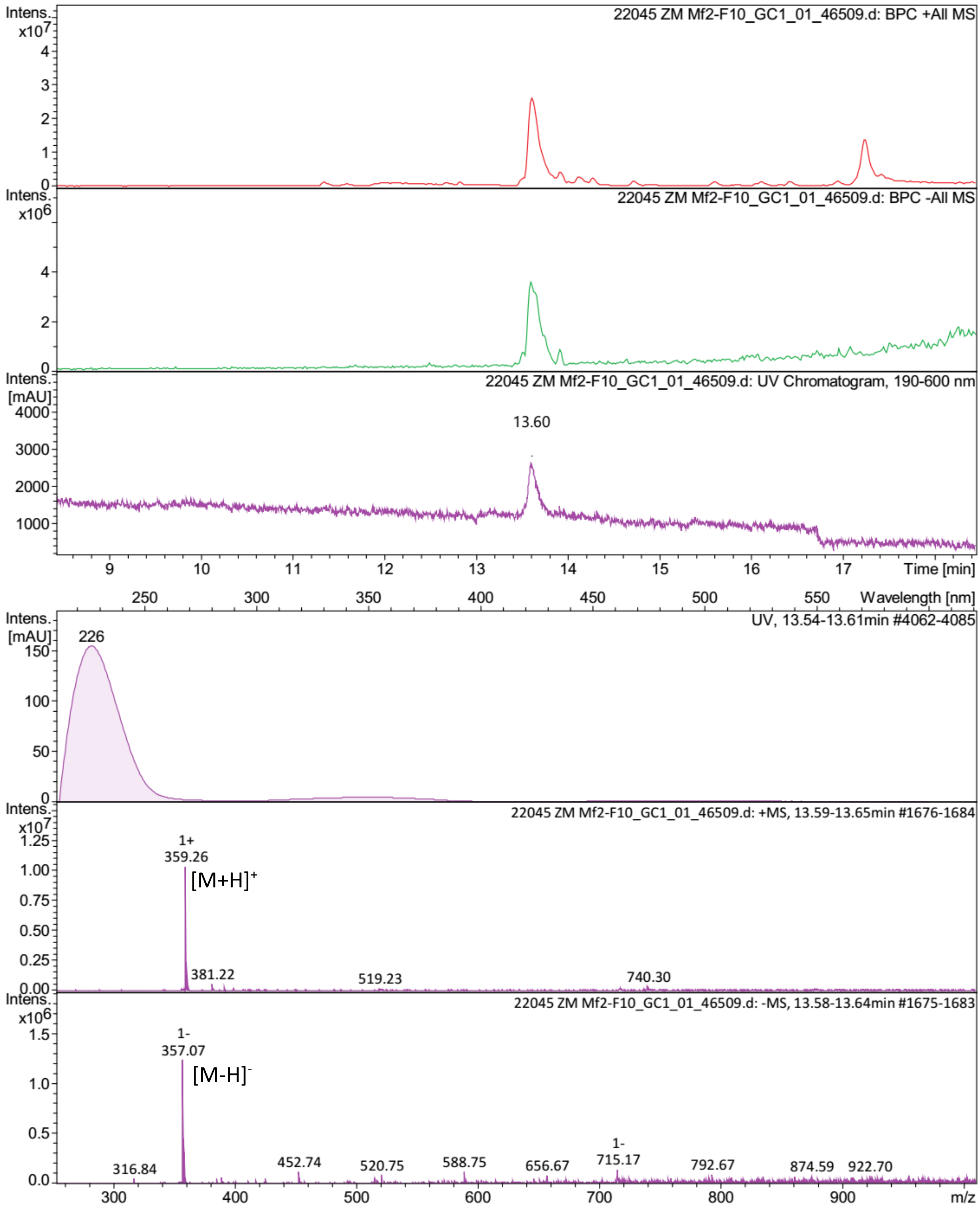

Bruker Compass DataAnalysis 4.4 printed: 14.07.2023 09:59:17 by: sel22 Page 1 of 1

Figure S9. HPLC-LRESIMS of **2**.

Generic Display Report

Analysis Info

Analysis Name	S:\DATA\MaXis\GPH22_Gift_Kunthide_Phutthacharoen\23_05\22045 ZM MF2-F10_16_01_11432.d	Acquisition Date	03.05.2023 13:31:32
Method	pos_säure_10000_screening_ms_100_2500_line.m	Operator	ate06
Sample Name	22045 ZM MF2-F10	Instrument	maXis
Comment	Screening01<br>Waters Acquity UPLC BEH C_18 1,7um 2.1x50mm		

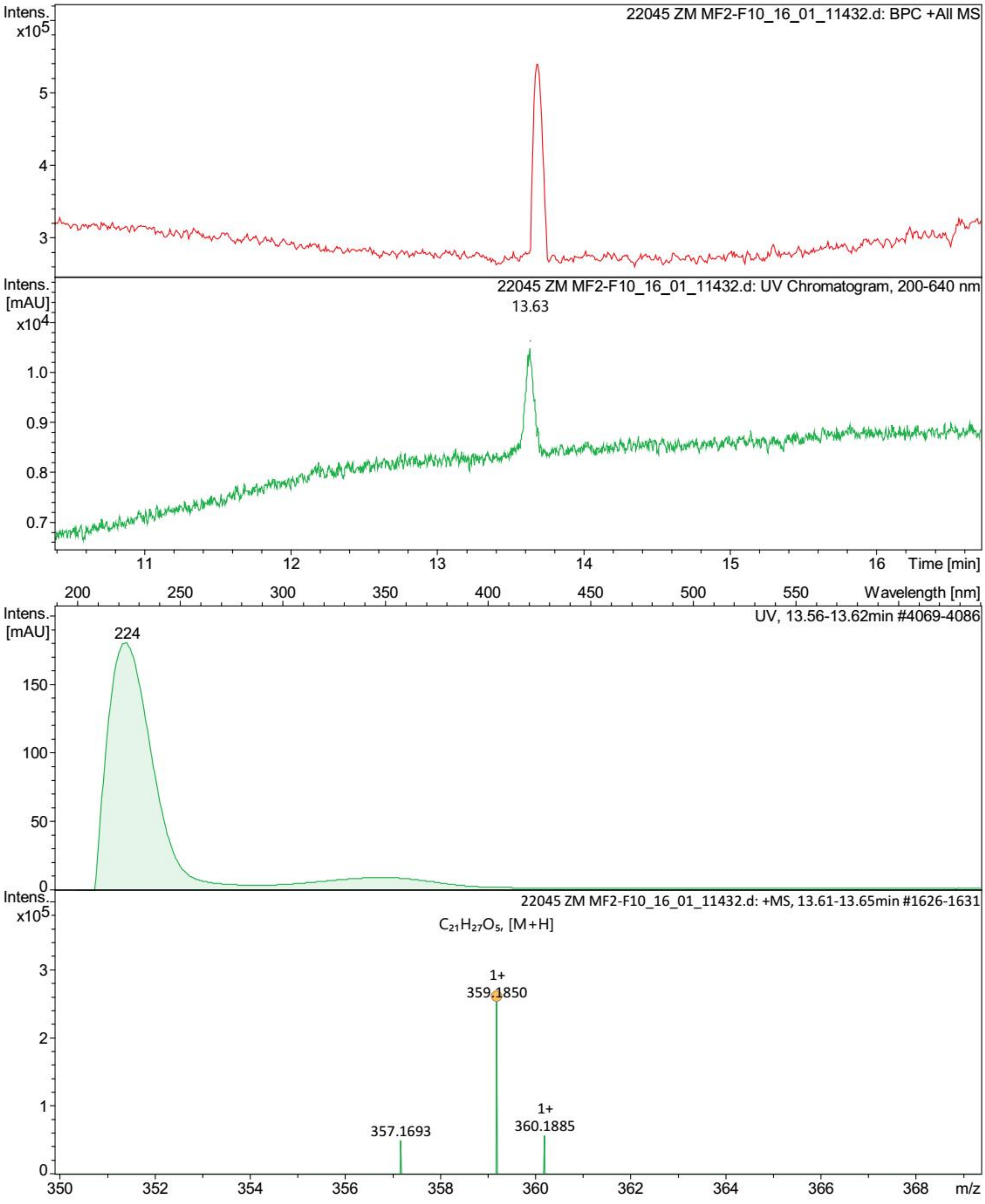

Bruker Compass DataAnalysis 4.4 printed: 14.07.2023 09:47:36 by: sel22 Page 1 of 1

Figure S10. HPLC-HRESIMS of **2**.

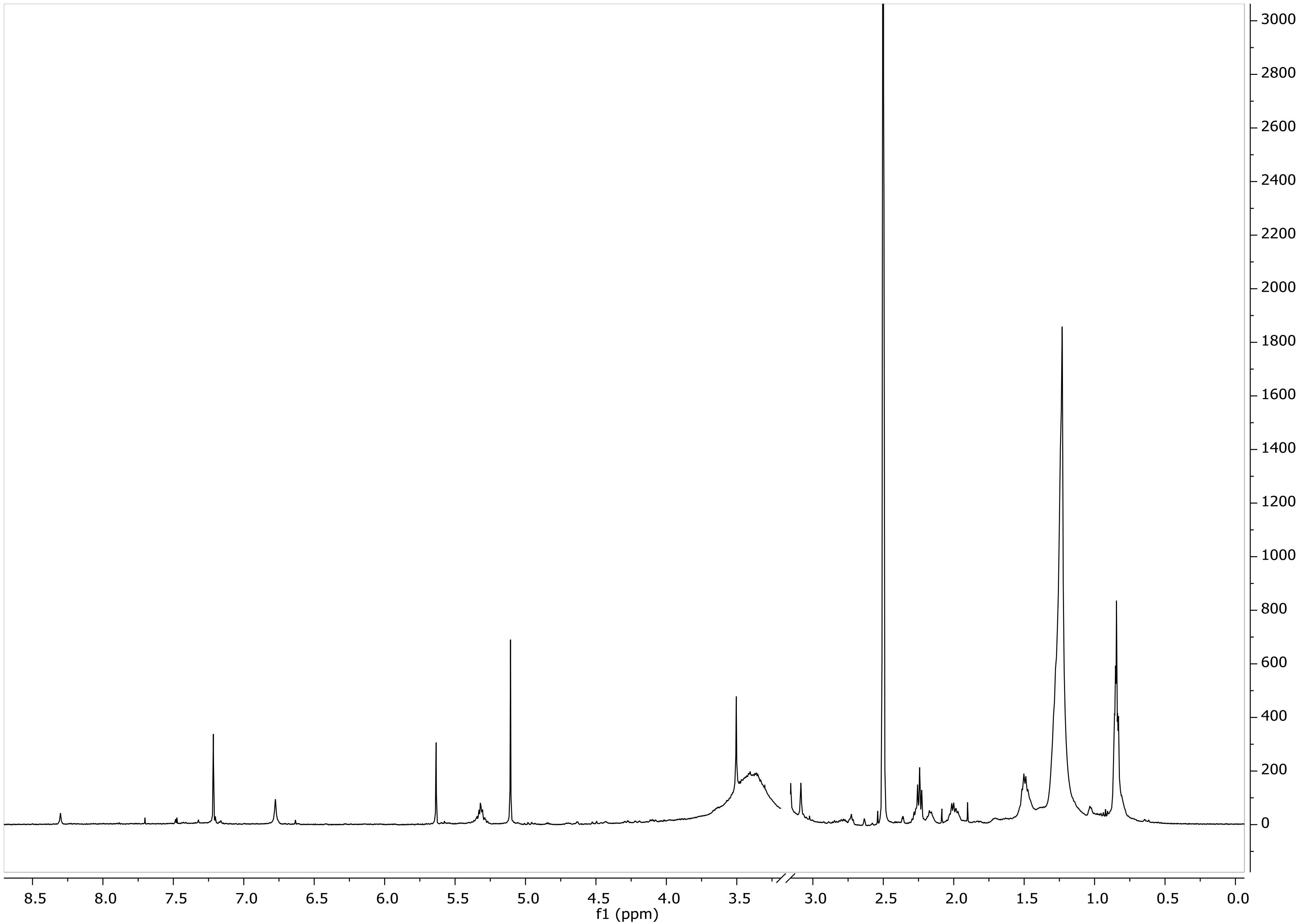

Figure S11. ^{1}H NMR spectrum of **2** in DMSO-d_6 at 500 MHz.

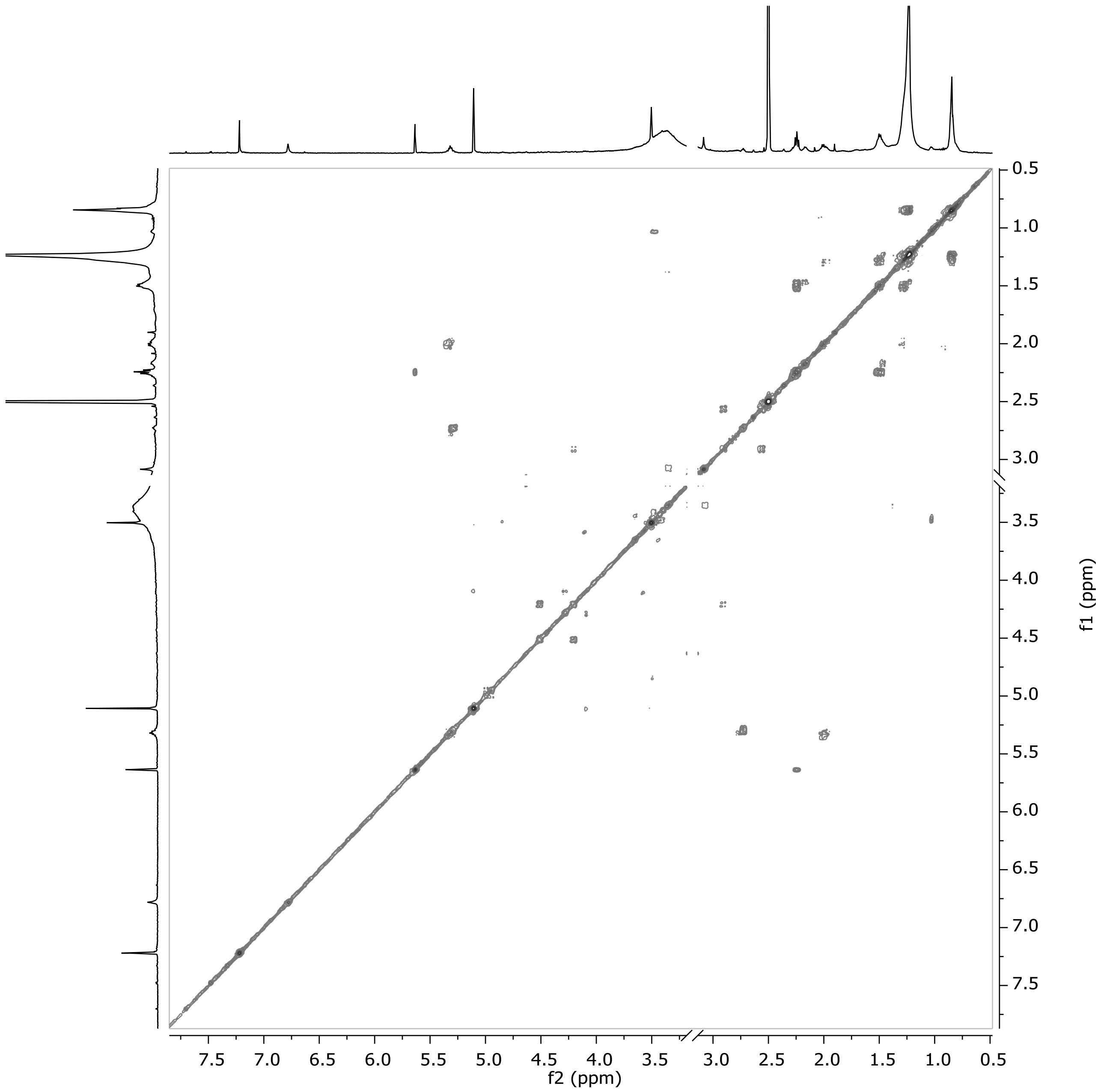

Figure S12. ^{1}H-^{1}H COSY spectrum of **2** in DMSO-d_6 at 500 MHz.

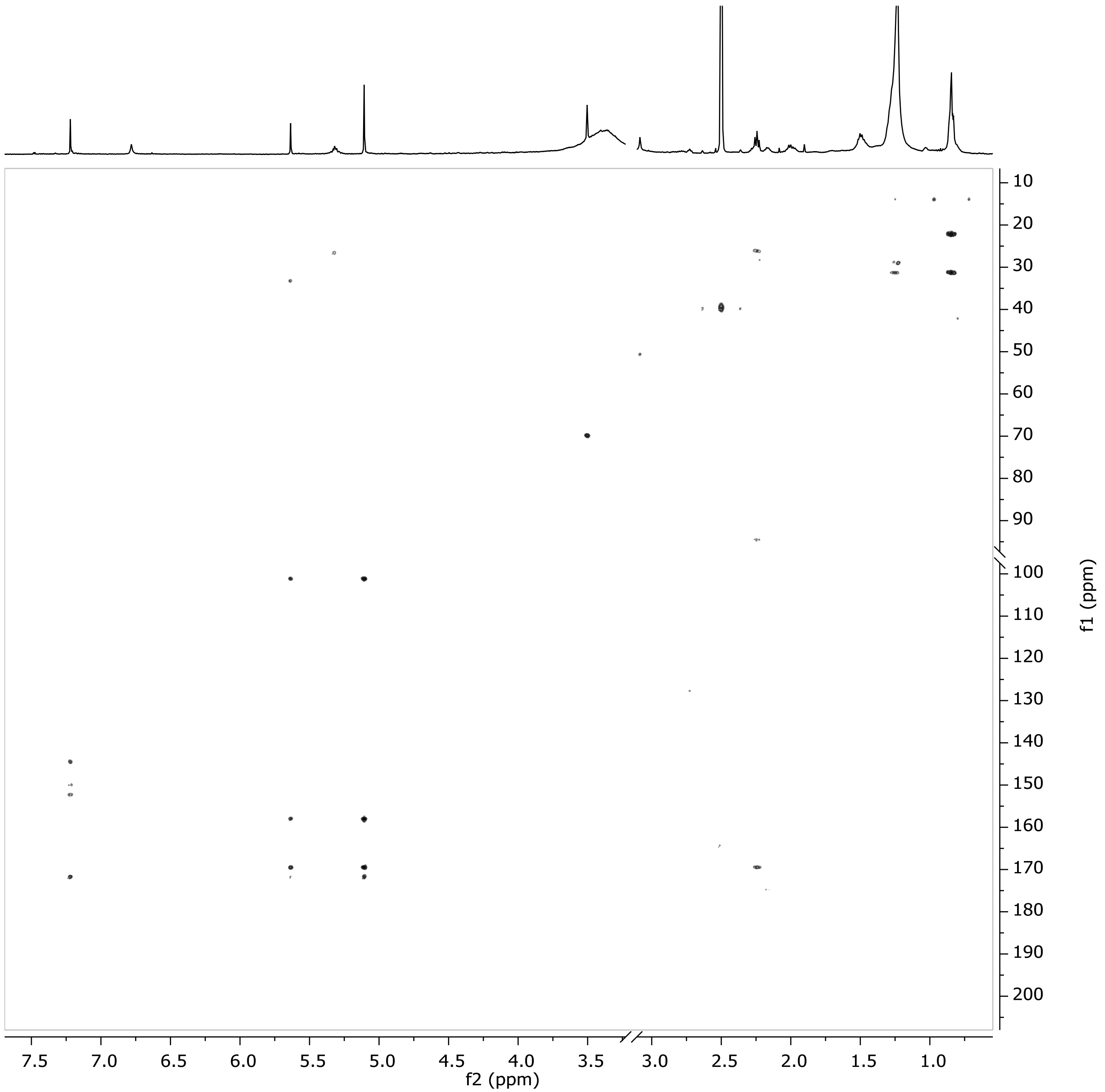

Figure S13. HMBC spectrum of **2** in DMSO-d_6 at 500 MHz.

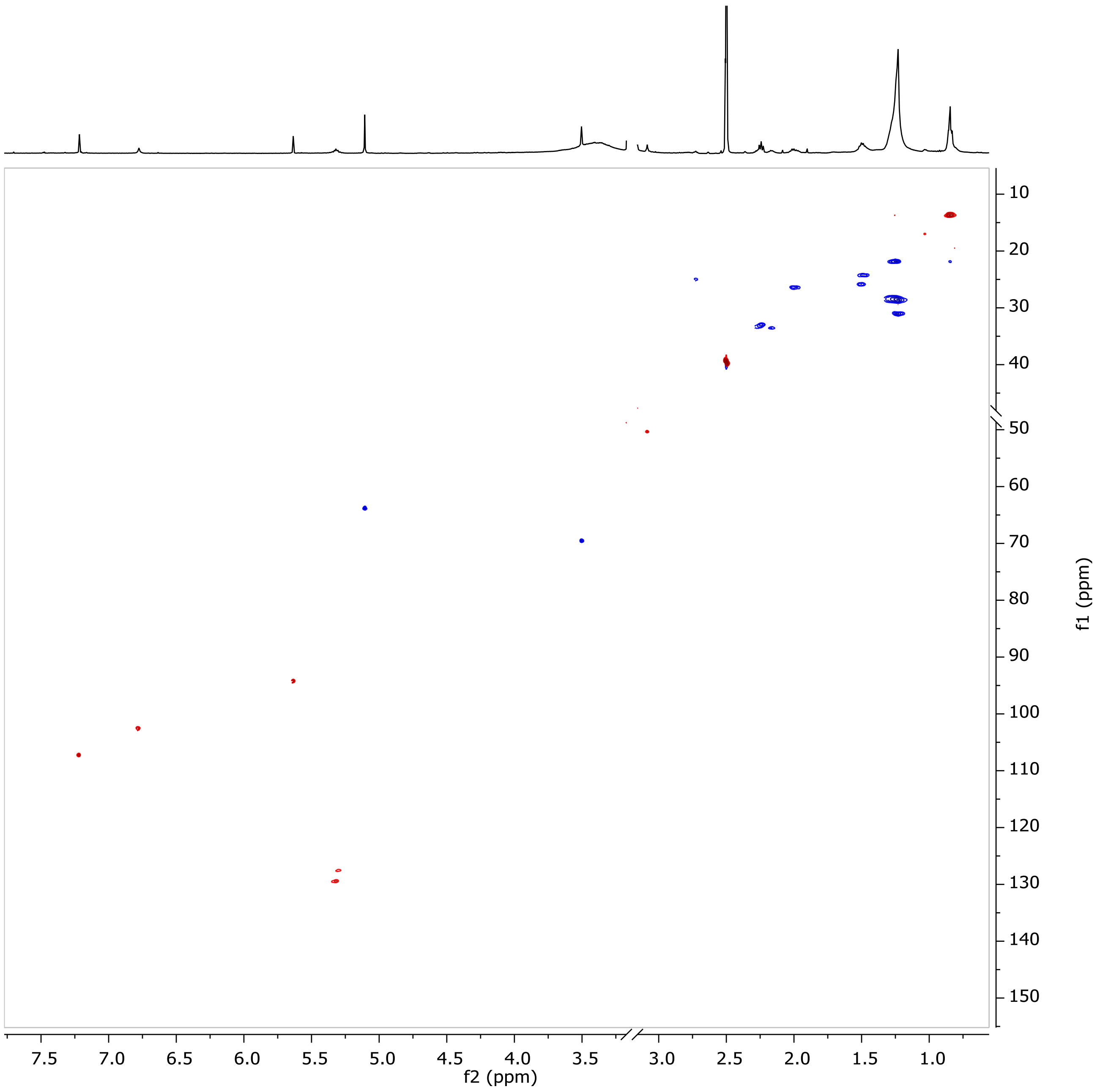

Figure S14. HSQC spectrum of **2** in DMSO-d_6 at 500 MHz.

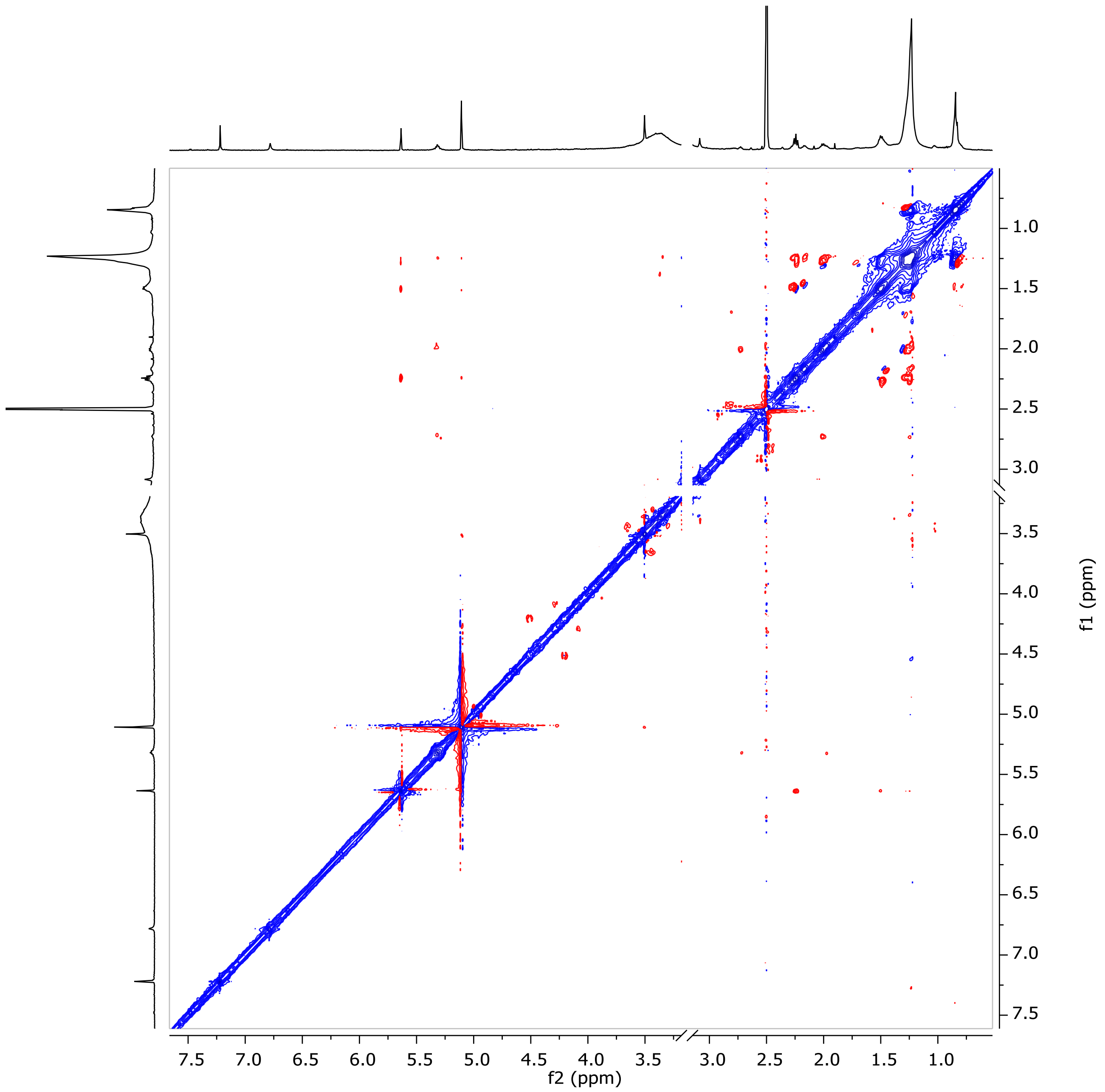

Figure S15. ROESY spectrum of **2** in DMSO-d_6 at 500 MHz.

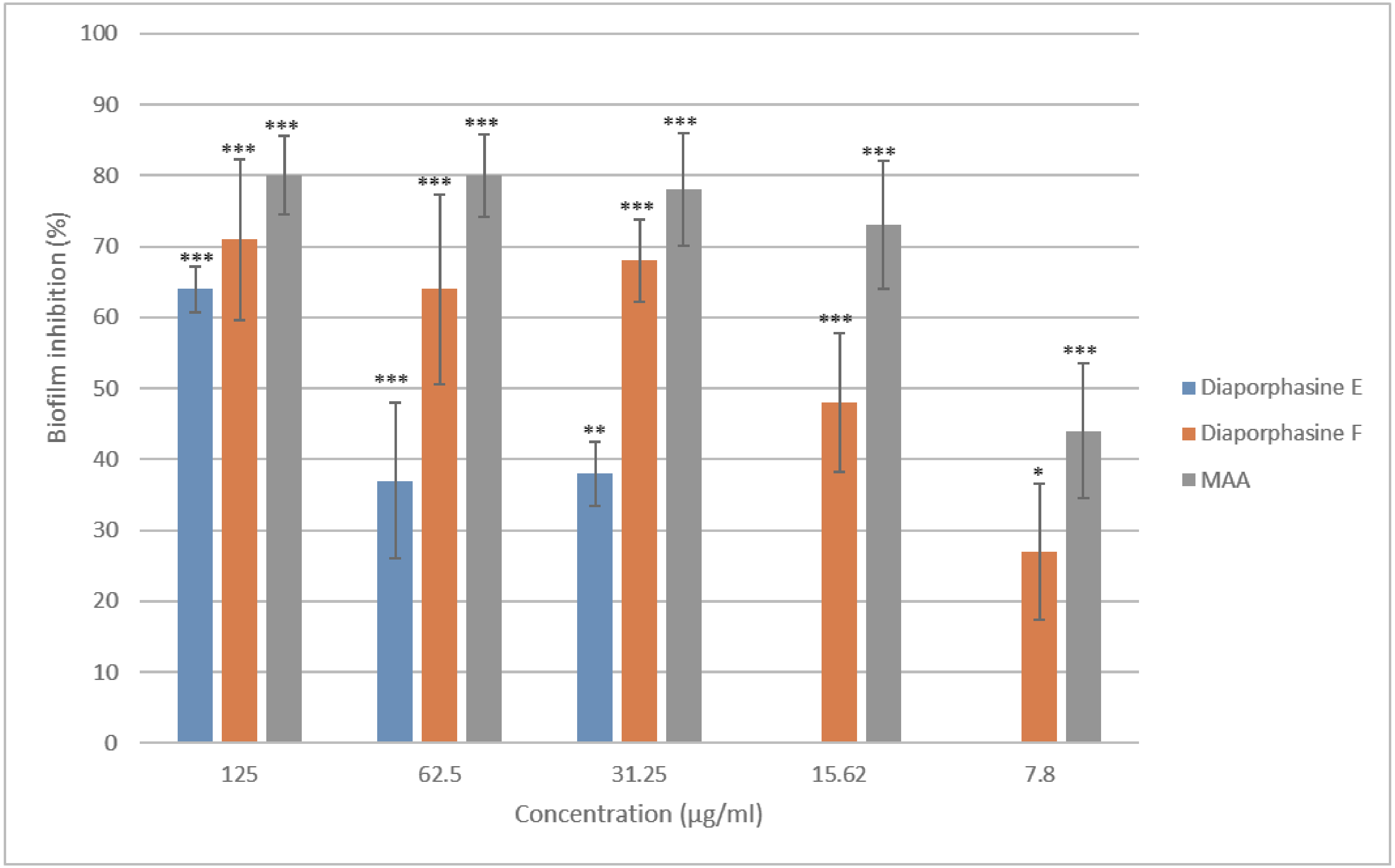

Figure S16. Effects on the biofilm formation of *S. aureus* after 24 h treatment with diaporphasines E (**1**) and F (**2**). Microporenic acid A (MAA) was used as positive control. Methanol was used as a solvent control and taken as 100%. Error bars indicate standard deviation of duplicates in two biological repeats; p values: * $p < 0.05$, ** $p < 0.01$, *** $p < 0.001$.

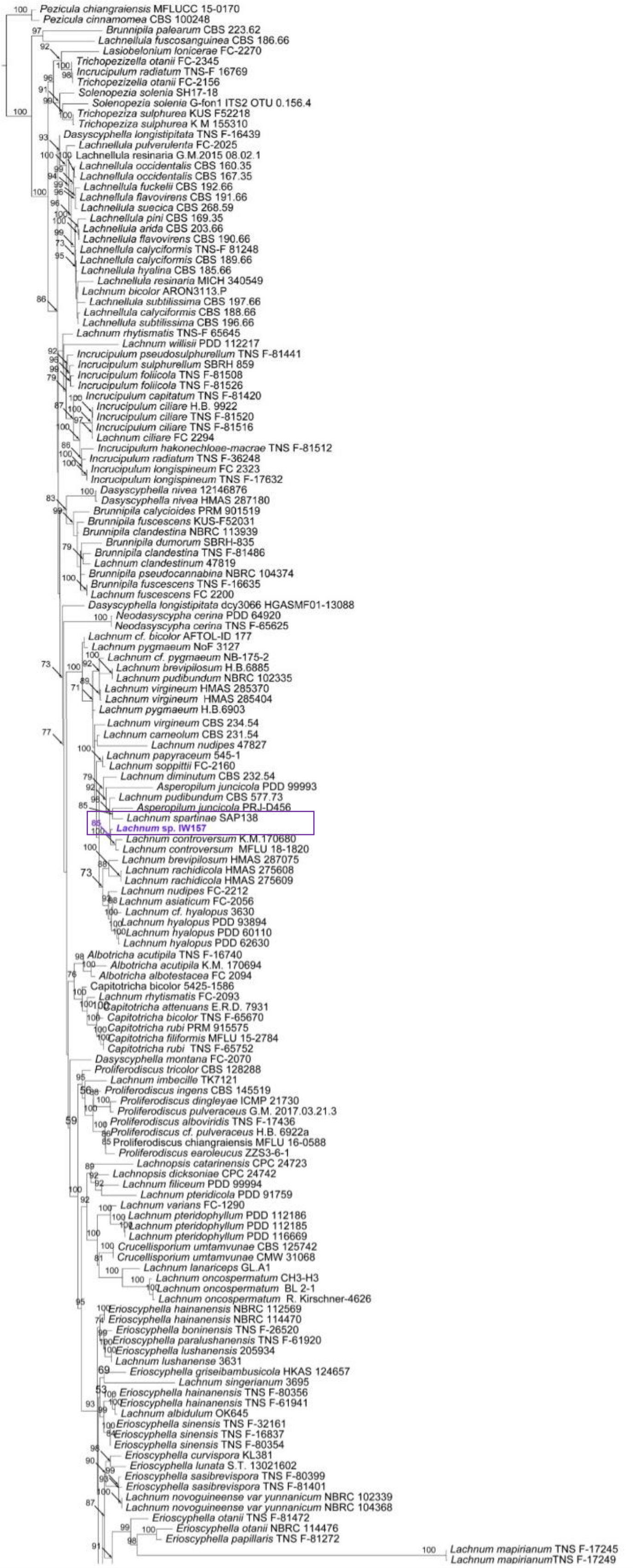

Figure S17. Maximum likelihood phylogenetic tree inferred from 177 taxa of Lachnaceae based on combined LSU and ITS sequence data. MLBP values ≥70% are given above the nodes. Strain/culture numbers are given after the taxon names. The tree is rooted with *Pezicula chiangraiensis* MFLUCC 15-0170 and *P. cinnamomea* CBS 100248. Newly generated sequence is in **purple**.

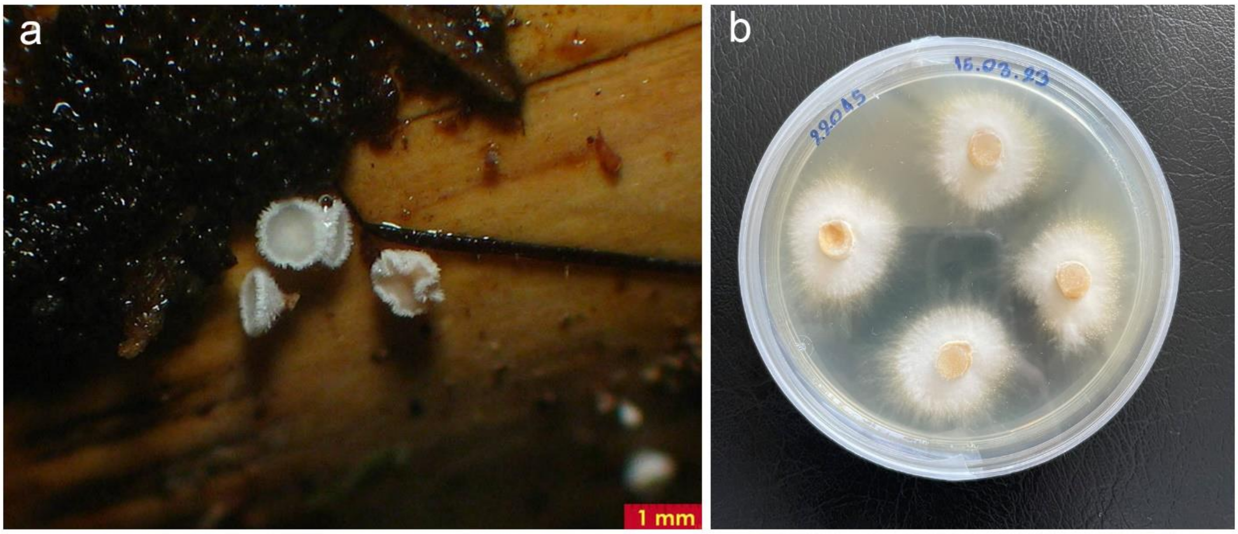

Figure S18. a. *Lachnum* sp. IW157 growing on *Phragmites communis* (Poaceae) b. Culture of *Lachnum* sp. IW157 on YM media

Table S1. Minimum inhibitory concentration (MIC) test for crude extract of *Lachnum* sp. IW157 (DSM 116717) against *Escherichia coli*, *Bacillus subtilis* and *Candida tenuis*.

	DSM 116717 in media	MIC (µg/mL)		
		E. coli	*B. subtilis*	*C. tenuis*
1	YM-S	300 µg/mL	300 µg/mL	-
2	YM-M	300 µg/mL	300 µg/mL	-
3	Q6-S	-	-	-
4	Q6-M	-	-	-
5	ZM-S	-	-	-
6	ZM-M	75 µg/mL	75 µg/mL	300 µg/mL
7	MeOH*			
8	Antibiotics**	Ciprofloxacine (1 mg/mL)	Penicillin (1 mg/mL)	Nystatin (4 mg/mL)

S: Supernatant extract. M: Mycelial extract.
*MeOH is a negative control. **Antibiotics is a positive control.

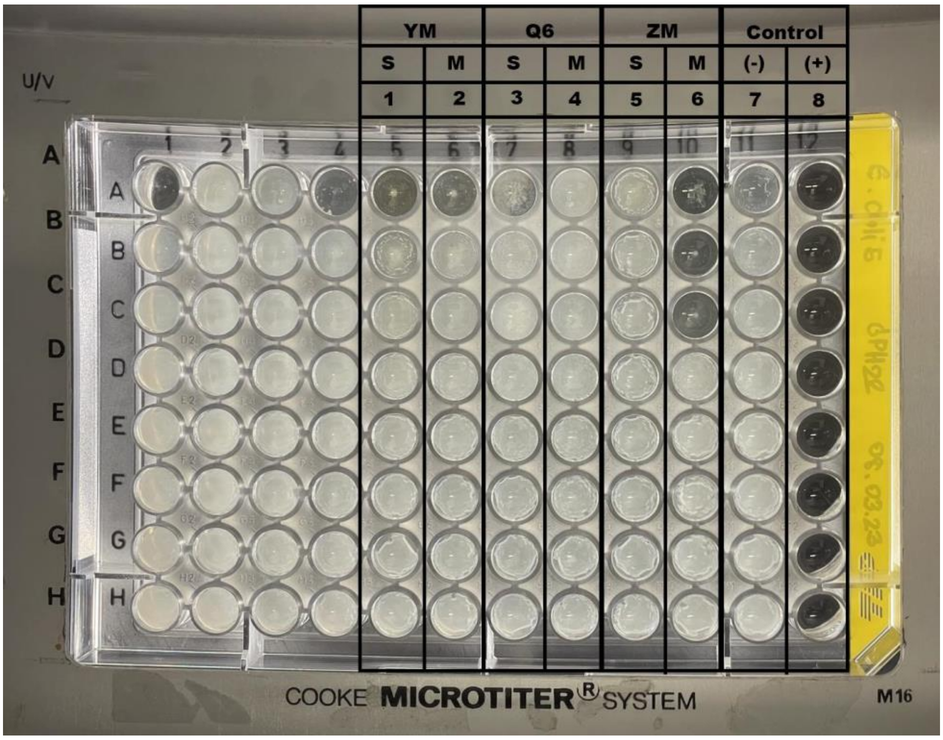

Figure S19. Shown MIC result of *Lachnum* sp. IW157 (DSM 116717) against *E. coli*.

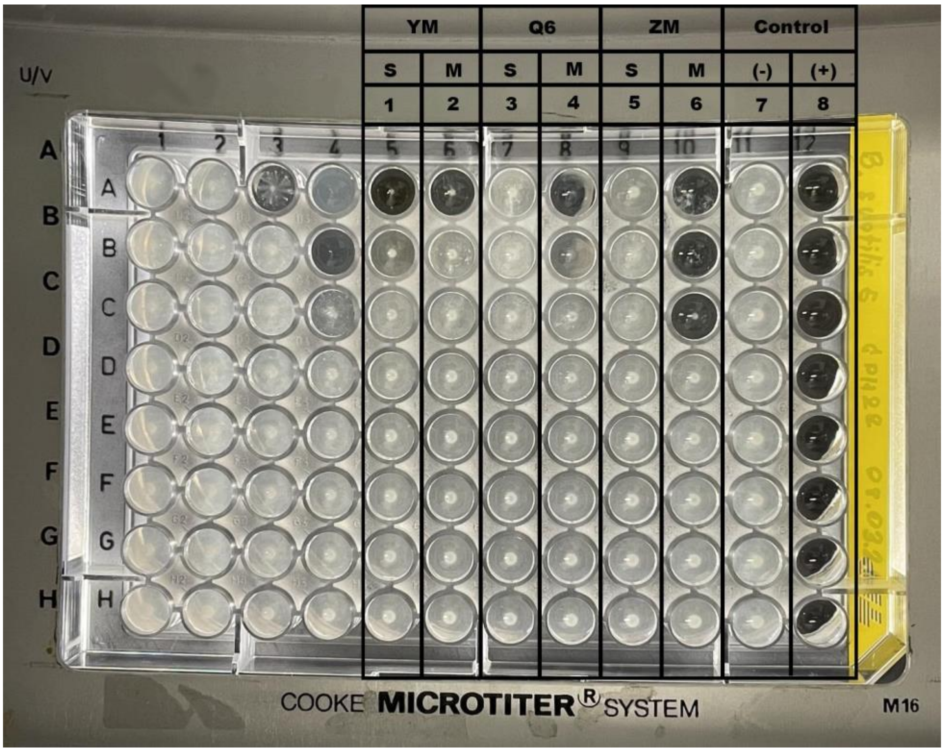

Figure S20. Shown MIC result of *Lachnum* sp. IW157 (DSM 116717) against *B. subtilis*.

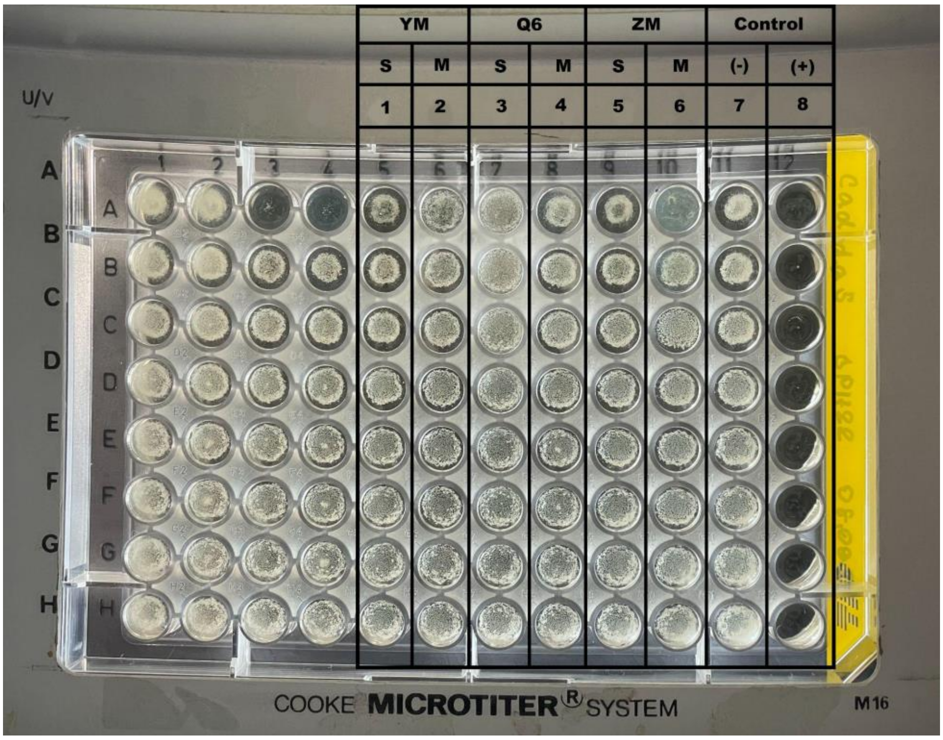

Figure S21. Shown MIC result of *Lachnum* sp. IW157 (DSM 116717) against *Candida tenuis*.

Table S2. Minimum inhibitory concentration (MIC) of **1** and **2** against test organisms.

**MIC (µg/mL)**			
**Test Organism**	**1**	**2**	**Reference**
Staphylococcus aureus DSM 346	n.i.	33.3	0.21 [G]
Bacillus subtilis DSM 10	n.i.	33.3	16.6 [O]
Escherichia coli DSM 1116	n.i.	n.i.	0.83 [G]
Mycobacterium smegmatis DSM ATCC700084	n.i.	n.i.	1.7 [K]
Pseudomonas aeruginosa DSM PA14	n.i.	n.i.	0.21 [G]
Candida albicans DSM 1665	n.i.	n.i.	4.2 [N]
Mucor hiemalis DSM 2656	n.i.	n.i.	4.2 [N]

n.i.: no inhibition observed, [G]: Gentamycin 1 mg/mL, [O]: Oxytetracyclin 1 mg/mL, [K]: Kanamycin 1 mg/mL, , [N]: Nystatin 10 mg/mL.
